# Genome-wide differential expression profiling of long non-coding RNAs in FOXA2 knockout iPSC-derived pancreatic cells

**DOI:** 10.1186/s12964-023-01212-2

**Published:** 2023-09-05

**Authors:** Ahmed K. Elsayed, Nehad M. Alajez, Essam M. Abdelalim

**Affiliations:** 1 Diabetes Research Center (DRC), Qatar Biomedical Research Institute (QBRI), Hamad Bin Khalifa University (HBKU), Qatar Foundation (QF), PO Box 34110, Doha, Qatar; 2 Stem Cell Core, Qatar Biomedical Research Institute (QBRI), Hamad Bin Khalifa University (HBKU), Qatar Foundation (QF), PO Box 34110, Doha, Qatar; 3 College of Health and Life Sciences, Hamad Bin Khalifa University (HBKU), Qatar Foundation (QF), Doha, Qatar; 4 Translational Cancer and Immunity Center (TCIC), Qatar Biomedical Research Institute (QBRI), Hamad Bin Khalifa University (HBKU), Qatar Foundation (QF), PO Box 34110, Doha, Qatar

**Keywords:** β-cell development, lncRNAs, Epigenetic, mRNA profile, Pancreatic islets

## Abstract

**Background:**

Our recent studies have demonstrated the crucial involvement of FOXA2 in the development of human pancreas. Reduction of FOXA2 expression during the differentiation of induced pluripotent stem cells (iPSCs) into pancreatic islets has been found to reduce α-and β-cell masses. However, the extent to which such changes are linked to alterations in the expression profile of long non-coding RNAs (lncRNAs) remains unraveled.

**Methods:**

Here, we employed our recently established FOXA2-deficient iPSCs (*FOXA2*^*−/−*^ iPSCs) to investigate changes in lncRNA profiles and their correlation with dysregulated mRNAs during the pancreatic progenitor (PP) and pancreatic islet stages. Furthermore, we constructed co-expression networks linking significantly downregulated lncRNAs with differentially expressed pancreatic mRNAs.

**Results:**

Our results showed that 442 lncRNAs were downregulated, and 114 lncRNAs were upregulated in PPs lacking FOXA2 compared to controls. Similarly, 177 lncRNAs were downregulated, and 59 lncRNAs were upregulated in islet cells lacking FOXA2 compared to controls. At both stages, we observed a strong correlation between lncRNAs and several crucial pancreatic genes and TFs during pancreatic differentiation. Correlation analysis revealed 12 DE-lncRNAs that strongly correlated with key downregulated pancreatic genes in both PPs and islet cell stages. Selected DE-lncRNAs were validated using RT-qPCR.

**Conclusions:**

Our data indicate that the observed defects in pancreatic islet development due to the FOXA2 loss is associated with significant alterations in the expression profile of lncRNAs. Therefore, our findings provide novel insights into the role of lncRNA and mRNA networks in regulating pancreatic islet development, which warrants further investigations.

Video Abstract

**Supplementary Information:**

The online version contains supplementary material available at 10.1186/s12964-023-01212-2.

## Introduction

FOXA2 has distinct functions in various tissues. In pancreatic development, FOXA2 is expressed early on, starting from the endoderm stage, with its protein level increasing during endocrine specification [1, 2]. On the other hand, exocrine and ductal cell express FOXA2 at low levels [1]. We have recently reported that FOXA2 plays critical roles in human pancreatic and hepatic development, using human induced pluripotent stem cells (iPSCs) [3, 4]. FOXA2 is known to regulate the expression of multiple transcription factors (TFs) that control pancreatic endocrine cell fate and insulin secretion [5, 6]. Recent genomic studies have shown that FOXA2-bound enhancers in humans are associated with type 2 diabetes (T2D) risk alleles [7]. Additionally, a recent study reported a patient with diabetes caused by a heterozygous missense variant in FOXA2 [8]. These findings highlight the potential contribution of FOXA2 defects to the development of diabetes and its crucial role in pancreatic endocrine differentiation.

Earlier studies have revealed that noncoding RNAs (ncRNAs) have regulatory roles both at the transcriptional and posttranscriptional levels [9, 10]. The long ncRNAs (lncRNAs) and epigenetic modifications are a crucial part of the transcriptional mechanisms that control cell specification and development [11]. LncRNAs play a significant role in regulating the expression of target genes, making them essential epigenetic modulators in various cell types [12]. These lncRNAs are distributed in the genome in a way that allows them to co-regulate the targeted protein-coding regions they regulate [13–15]. The cell-type specific expression pattern of lncRNAs suggests their potential role as mediators of lineage specification [16, 17]. Understanding the regulatory role of lncRNAs during pancreatic development is crucial in decoding the regulatory network controlling pancreatic islet development [18, 19]. Several specific lncRNAs in pancreatic islets have been mapped close to TFs that regulate pancreatic embryogenesis and β-cell development. Over 1000 lncRNAs have been identified in human pancreatic islets through integrative epigenetic analysis, indicating their importance in the endocrine and β-cell differentiation program during pancreatic development [20]. Various TFs, such as PDX1, NKX6.1, NKX2.2, PAX6, and GATA6, that regulate pancreatic islet development have been identified to be regulated by lncRNAs such as PLUTO, MALAT1, ROIT, Gm10451, HI-LNC15, Paupar, PAX6-AS, and GATA6-AS [14, 21–27]. Given the lack of functional information about lncRNAs, stage-specific analyses throughout development are essential [11], especially considering the identification of lncRNAs specific to α-and β-cells in human islets [28]. Differential expression of lncRNAs has been reported in mouse islets with both type 1 diabetes (T1D) and type 2 diabetes (T2D) [29, 30], as well as in the islets of patients with T2D [20, 22]. These findings support the potential for lncRNAs to play a vital role in the development and functionality of pancreatic β-cells, and suggest that they may serve as biomarkers for early diagnosis, as has been reported in blood samples from T1D patients [31].

Previous studies have shown that FOXA2 expression is regulated by various lncRNAs, including *lncRNA-NEF* [32, 33], *lncRNA-FTX* [34], *lncRNA-HOTAIR* [35], and *lncRNA-Falcor* [36], with their overexpression leading to the activation of FOXA2 expression and their inhibition resulting in reduced FOXA2 expression. A previous study demonstrated that during human endoderm differentiation, FOXA2 is activated by lncRNA DEANR1 (*LINC00261*) [37], which has been shown to be essential for generation of insulin + cells from hESCs [38]. Interestingly, a regulatory feedback loop has been identified between FOXA2 and its associated lncRNAs, with studies in lung tissue indicating that FOXA2 can either activate or repress its regulatory lncRNAs. FOXA2 binds to the promotor of *lncRNA-Falcor,* leading to its repression [36], while it activates the expression of *lncRNA-NEF,* a direct downstream target of FOXA2 [33]*.* The function of lncRNAs during pancreatic lineage specification is not fully understood. Our recent studies revealed that the expression of several genes involved in the development and function of pancreatic islet cells is dysregulated by FOXA2 deficiency [3, 39]. Therefore, in the current study, we used established *FOXA2*^*−/ −*^iPSC lines to investigate the effect of FOXA2 loss on the lncRNA profiles in the pancreatic progenitors (PPs) and pancreatic islets derived from hiPSCs.

## Materials and methods

### Differentiation of iPSCs into pancreatic progenitors and pancreatic islets

Two different FOXA2 knockout iPSC lines (*FOXA2*^*−/−*^iPSCs) recently established in our lab were used in this study [3]. Both *FOXA2*^*−/−*^iPSC lines and their isogenic controls (WT-iPSCs), were differentiated into PPs and pancreatic islets using our modified stepwise differentiation protocol [3] as illustrated in Supplementary Fig. 1A.

### RNA extraction and qPCR analysis

RNeasy Plus Mini Kit (QIAGEN) used for total RNA extraction following the manufacturer's instructions. The RNA was reversely transcribed using superscript IV, First-Strand Synthesis System (Thermo Fisher Scientific). The quantity and integrity of RNA quantity were assessed using Agilent Bioanalyzer 2100 (Agilent Technologies). The sequences of selected lncRNA primers listed in Supplementary Table 1. The amplification was detected using Quant Studio 7 system (Applied Biosystems) using GoTaq qPCR Master Mix (Promega) and GAPDH as an internal control. Fold change 2-^ΔΔ Ct^ used to present the expression level of performed lncRNAs.

### Total RNA library preparation and sequencing

RNA was extracted using Direct-zol RNA extraction kit (Zymo Research) from two biological replicates for each sample of cells at PPs and pancreatic islets stages of differentiation. mRNA was captured from 1 μg of total RNA using NEBNext (Poly A) mRNA magnetic isolation kit (NEB, E7490) according to the manufacturer’s instructions. NEBNext ultra directional RNA library prep kit (NEB, E7420L) used to NEBNext ultra directional RNA library prep kit (NEB, E7420L) used to prepare RNA-seq libraries which is sequenced on an Illumina Hiseq 4000 system. The initial processing of the raw data involved basic trimming and quality control, which was carried out using Illumina BCL2Fastq Conversion Software v2.20.

### Total RNA‑Seq data and bioinformatics analysis

Pair-end FASTQ files were subsequently aligned to the GRCh38 reference genome using built-in module and default settings in CLC genomics workbench v21.0.5. Normalized expression data (TPM, transcript per million) were then subjected to differential expression analysis using twofold change (Log2 FC 1) and < 0.05 p-value cut-off. Transcripts with raw expression values < 1.0 TPM were excluded from the analysis. Differential expression analysis and hierarchical clustering were conducted using AltAnalyze v.2.1.3 as described before [40, 41].

### Correlation analysis between lncRNA and differentially expressed genes (DEGs)

To construct networks between differentially expressed genes (DEGs) and corresponding DE-lncRNAs in the context of FOXA2 loss, we first identified the list of DEGs essential for PP development, and the corresponding DE-lncRNAs using transcriptome analysis of iPSC-derived PPs (stage 4 of differentiation), and iPSC-derived pancreatic islets (stage 7 of differentiation) from *FOXA2*^*−/−*^ iPSCs and WT controls. We subsequently assessed the relevance of identified networks to normal pancreatic cell development, by retrieving the mRNA and lncRNA expression data from 305 normal pancreatic tissue samples from the Genotype-Tissue Expression (GTEx) portal (https://gtexportal.org/home/). Correlation between the identified DE-lncRNAs-DEGs based on the in vitro iPSC-derived PPs, and iPSC-derived pancreatic islets were subsequently validated in the GTEx pancreatic dataset. Pearson correlation analysis was performed on the expression values of DE-lncRNA and DEG pairs using IBM SPSS statistics v26. We selected the co-expressed pairs (LncRNA-DEG) with a Pearson correlation coefficient ≥ 0.3 to establish and draw the network using Cytoscape software (National Resource for Network Biology), as described before [42, 43].

### Statistical analysis

Statistical analysis was performed using unpaired two-tailed student’s t-test by Prism 8 software, with data represented as mean ± standard deviation (SD).

## Results

### Generation of pancreatic progenitors and islets from FOXA2 ^*–/–*^ iPSCs and wild-type iPSCs

To evaluate the influence of FOXA2 deficiency on lncRNA expression profiles in PPs and pancreatic islets, we differentiated *FOXA2*^*–/–*^ iPSC lines into PPs and pancreatic islets following our previously described protocol [3] (Supplementary Fig. 1A). Lack of FOXA2 resulted in a significant decrease in the expression levels of the crucial pancreatic progenitor markers, PDX1 and NKX6.1 (Supplementary Fig. 1B), as recently reported in our published article [3]. The absence of FOXA2 resulted in a substantial decrease in the expression levels of endocrine progenitor markers, including NGN3 and NKX2.2 (Supplementary Fig. 1C). In addition, there was an almost complete loss of insulin (INS) and glucagon (GCG) expression indicating a reduction in the masses of β-cells and α-cells (Supplementary Fig. 1D). The complete loss of FOXA2 protein was confirmed through western blotting, as we previously reported [3, 39]. These findings validate our previously published results regarding the impact of FOXA2 absence on islet development [3, 39].

### Characterization of lncRNA profiles in FOXA2 knockout iPSC-derived pancreatic progenitors

**Fig. 1:**
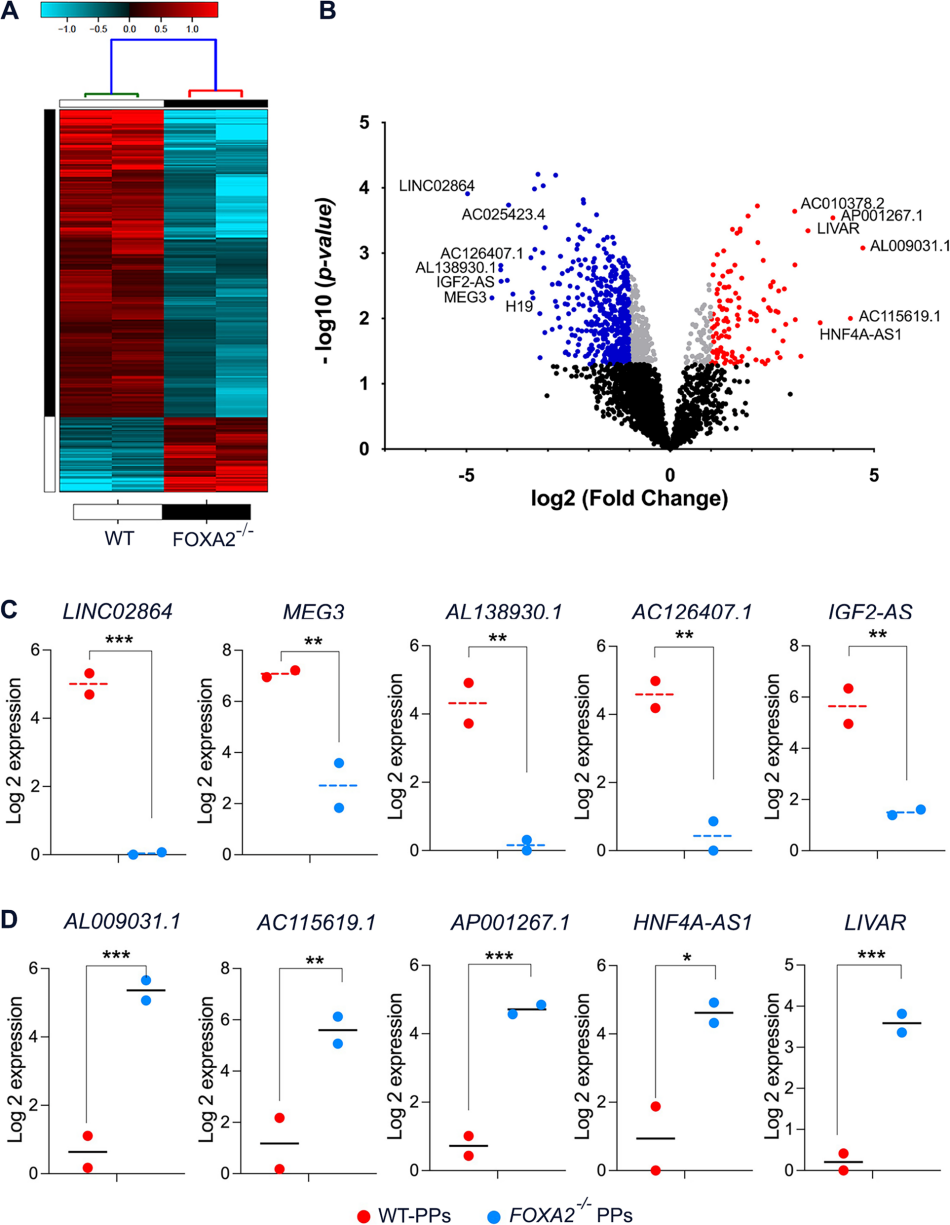
Differentially expressed lncRNAs in pancreatic progenitors (PPs) derived from *FOXA2*^*−/−*^ iPSCs compared to those derived from WT-iPSCs. **A** Hierarchical clustering of the captured lncRNAs in the pancreatic progenitors (PPs) from FOXA2 knocked out iPSCs (*FOXA2*^*−/−*^ iPSCs) and WT-iPSCs. Each column represents one differentiation experiment, and each row represents the lncRNA transcript. The expression level of each transcript (log2) is depicted according to the color scale. **B** Volcano plot depicting upregulated (red) and downregulated (blue) lncRNAs (*p* < 0.05, Log2 FC > 1). Graphs represent the expression of the top 5 downregulated (**C**) and upregulated (**D**) lncRNA transcripts

To assess the impact of FOXA2 loss on the expression profile of lncRNAs in PPs, we conducted RNA-Seq analysis on *FOXA2*^*–/–*^PPs and WT-PPs. We identified a total of 826 DE-lncRNAs in *FOXA2*^*–/–*^ PPs compared to WT-PPs, with 442 significantly downregulated (Log2 FC <  − 1.0, *p* < 0.05) and 114 significantly upregulated (Log2 FC > 1.0, *p* < 0.05) DE-lncRNAs (Fig. 1A and Supplementary Table 2). Figure 1B presents the volcano plot of the DE-lncRNAs in *FOXA2*^*–/–*^ PPs versus WT-PPs. The expression of the top 5 upregulated and top 5 downregulated lncRNA transcripts illustrated in Fig. 1C and D, with *LINC02864* being the most significantly downregulated and *AL009031.1* being the most significantly upregulated lncRNA transcripts in *FOXA2*^*–/–*^ PPs compared to WT-PPs.

### Correlations between DE-lncRNAs and DE-mRNAs in iPSC-derived pancreatic progenitors

**Fig. 2:**
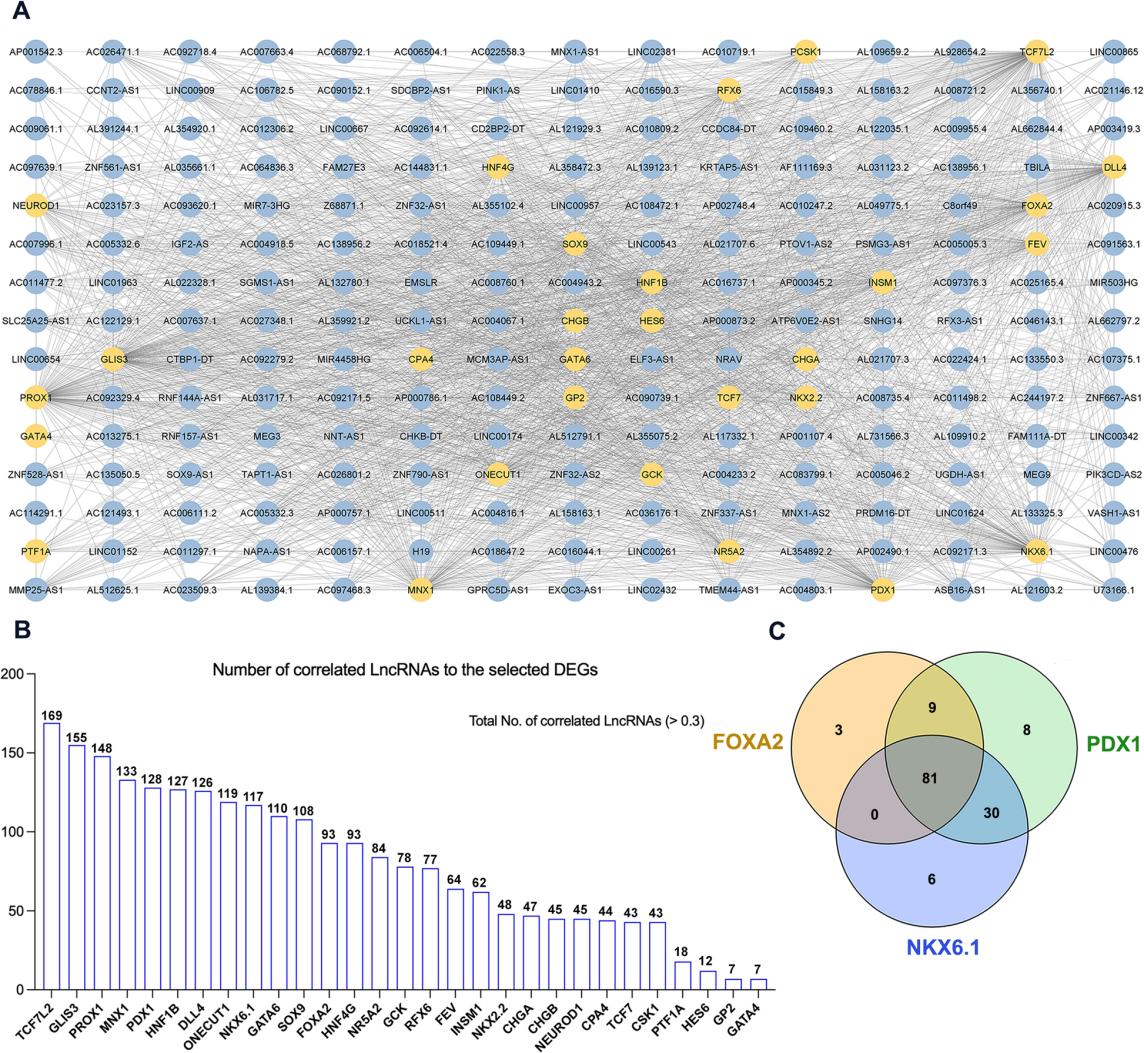
Co-expression network analysis of downregulated lncRNAs and DEGs in pancreatic progenitors derived from *FOXA2*^*−/−*^ iPSCs. The correlation analysis between the downregulated DE-lncRNAs and previously identified DEGs in our previous study of the pancreatic progenitor (PP) stage after its incorporation with the online database of 305 pancreatic tissues (**A**). The number of correlated lncRNAs with each specific DEG is presented in the graph (**B**), and (**C**) shows the commonly correlated lncRNAs with FOXA2, PDX1, and NKX6.1

Next, we sought to explore the potential function of the identified DE-lncRNAs in this study. To accomplish this, we conducted a correlation analysis to establish links between these DE-lncRNAs and selected DEGs (Supplementary Table 3) that are known to be essential for PPs, as reported in our recent study [39]. Our analysis identified significant correlation between numerous DEGs and lncRNAs, based on the *FOXA2*^*–/–*^PPs and WT-PPs in vitro models. To assess the relationship between the identified DEGs and lncRNAs from the in vitro model in normal pancreatic cells, we subsequently obtained mRNA and lncRNA expression data from an online database of 305 pancreatic tissues (https://gtexportal.org/home/). Our analysis revealed significant correlations between the downregulated DE-lncRNAs and key pancreatic genes that were also downregulated, based on the *FOXA2*^*–/–*^PPs and WT-PPs in vitro models. These genes include *PDX1, NKX6.1, FOXA2, RFX6, GATA6, GATA4, PTF1A, NEUROD1, NKX2.2, INSM1, FEV, DALL4, CPA2, ONECUT1, MNX1, GLIS3, PROX1, TCF7L2, HES6, NR5A2, PCSK1, HNF4G, CHGA, CHGB, GP2,* and *GCK* (Fig. 2A). DE-lncRNAs and DEGs with Pearson correlation coefficients > 0.3 were selected and the DE-lncRNA-mRNA correlation network analysis in PPs was constructed (Fig. 2A). We excluded 191 downregulated DE-lncRNAs, with weak correlations (less than 0.3) to any of the selected DEGs. Out of the strongly correlated downregulated 195 DE-lncRNAs, 93 DE-lncRNAs were strongly correlated (> 0.3) with FOXA2 and were enlisted in Table 1 with their correlated genes (Fig. 2B). Furthermore, we identified 169, 155, 148, 133, 128, 127, 126, 119, 117, and 110 DE-lncRNAs that strongly correlated with *TCF7L2*, *GLIS3*, *PROX1*, *MNX1*, *PDX1*, *HNF1B*, *DALL4*, *ONECUT1*, *NKX6.1*, and *GATA6*, respectively (Fig. 2B). PDX1 and NKX6.1 are known as the main TFs that mark the pancreatic precursors of β-cells. In our analysis, we identified 81 DE-lncRNAs that were commonly correlated with *FOXA2*, *PDX1,* and *NKX6.1* in normal pancreatic cells (Fig. 2C, Supplementary Fig. 2, Supplementary Table 4). The most downregulated DE-lncRNAs of this common list were *MEG3*, *H19*, *ZNF667-AS1*, *AC013275.1*, *LINC00543*, *LINC00261*, *MIR7-3HG*, *AC097639.1*, *PRDM16-DT*, *LINC02381*, *LINC01963*, *AL662797.2*, *LINC00511*, *AP000345.2*, *GPRC5D-AS1*, *NRAV*, and *MNX1-AS1* (Supplementary Fig. 2). We noticed that the top correlated DE-lncRNAs were significantly downregulated and were linked to FOXA2. It is intriguing to note that the results obtained when we integrated the DE-lncRNAs and DEGs of our RNA-Seq analysis were consistent with those obtained from normal pancreatic tissues, as shown in Supplementary Fig. 3.

**Table 1:** List of the strongly correlated lncRNAs to FOXA2 TF with Pearson correlation (PC) > 0.3 in the pancreatic progenitors (PPs) derived from *FOXA2*^−/−^iPSCs compared to those derived from WT-iPSCs (*p* < 0.05)

**LncRNA**	**PC**	**Log2-FC**	***P*-value**	**Other TFs correlated to the lncRNA**
**Downregulated**				
LINC00543	0.636**	-3.09914	0.001683	HNF1B, HNF4G, PDX1, NKX6-1, NKX2-2, GATA6, SOX9, HES6, MNX1, TCF7L2, PROX1, GLIS3, CHGA, CHGB,PCSK1, NR5A2
AC005332.6	0.567**	-1.16633	0.003212	ONECUT1, HNF1B, HNF4G, PDX1, NKX6-1, NKX2-2, NEUROD1,, GATA6, SOX9, HES6, MNX1, RFX6, TCF7L2, TCF7, PROX1, GLIS3, CPA4, CHGA, CHGB, FEV, PCSK1, DLL4, INSM1, NR5A2
MMP25-AS1	0.534**	-1.171816	0.028843	ONECUT1, HNF1B, HNF4G, PDX1, NKX6-1, NKX2-2,GATA6, SOX9, HES6, MNX1, RFX6, TCF7L2, TCF7, PROX1, GLIS3, PTF1A, CPA4, CHGA,PCSK1, DLL4, INSM1, NR5A2
MEG3	0.518**	-4.373715	0.004838	ONECUT1, HNF1B, HNF4G, PDX1, NKX6-1, GATA6, SOX9, MNX1, RFX6, TCF7L2, TCF7, PROX1, GLIS3, PTF1A, CPA4,FEV, PCSK1, DLL4, INSM1,NR5A2
NRAV	0.518**	-2.086095	0.001259	ONECUT1, HNF1B, HNF4G, PDX1, NKX6-1, NKX2-2, NEUROD1,, GATA6, SOX9, MNX1, RFX6, TCF7L2, TCF7, PROX1, GLIS3, CHGA, CHGB, FEV, PCSK1, DLL4, INSM1, NR5A2
ASB16-AS1	0.513**	-1.535975	0.049223	ONECUT1, HNF1B, HNF4G, PDX1, NKX6-1, NKX2-2, NEUROD1, GATA6, SOX9, HES6, MNX1, RFX6, TCF7L2, TCF7, PROX1, GLIS3, CHGA, CHGB, FEV, PCSK1, DLL4, INSM1, GCK, NR5A2
PTOV1-AS2	0.510**	-1.537089	0.028823	ONECUT1, HNF1B, HNF4G, PDX1, NKX6-1, NKX2-2, NEUROD1,, GATA6, SOX9, HES6, MNX1, RFX6, TCF7L2, TCF7, PROX1, GLIS3, CPA4, CHGA,, FEV, DLL4, INSM1, GCK, NR5A2
AC013275.1	0.504**	-3.199973	0.008419	ONECUT1, HNF1B, HNF4G, PDX1, NKX6-1, NKX2-2, NEUROD1, GATA6, SOX9,, MNX1, RFX6, TCF7L2, TCF7, PROX1, GLIS3, CPA4, CHGA, CHGB, FEV, PCSK1, DLL4, INSM1, GCK, NR5A2
AC012306.2	0.499**	-1.257744	0.031814	HNF1B, HNF4G, PDX1, NKX6-1, GATA6, SOX9, MNX1, TCF7L2, PROX1, GLIS3, NR5A2
AC007996.1	0.492**	-1.980823	0.000461	ONECUT1, HNF1B, HNF4G, PDX1, NKX6-1, NKX2-2, NEUROD1, GATA6, SOX9, MNX1, RFX6, TCF7L2, TCF7, PROX1, GLIS3, CPA4, CHGA, CHGB, FEV, PCSK1, DLL4, INSM1, GCK, NR5A2
AL021707.6	0.491**	-1.590208	0.011266	ONECUT1, HNF1B, HNF4G, PDX1, NKX6-1, GATA4, HES6, MNX1, TCF7L2, PROX1, GLIS3, PTF1A, CPA4, DLL4, NR5A2
CTBP1-DT	0.489**	-1.146042	0.026777	ONECUT1, HNF1B, HNF4G, PDX1, NKX6-1, NKX2-2, NEUROD1, GATA6, SOX9, MNX1, RFX6, TCF7L2, TCF7, PROX1, GLIS3, CHGA, CHGB, FEV, PCSK1, DLL4, INSM1, NR5A2
AC008760.1	0.489**	-1.059341	0.002437	ONECUT1, HNF1B, HNF4G, PDX1, NKX6-1, NKX2-2, NEUROD1, GATA6, SOX9, MNX1, RFX6, TCF7L2, TCF7, PROX1, GLIS3, PTF1A, CPA4, CHGA, CHGB, FEV, PCSK1, DLL4, INSM1, GCK, NR5A2
LINC00667	0.487**	-1.126357	0.040617	ONECUT1, HNF1B, HNF4G, PDX1, NKX6-1, NKX2-2, NEUROD1, GATA6, SOX9, MNX1, RFX6, TCF7L2, TCF7, PROX1, GLIS3, CHGA, CHGB, FEV, PCSK1, DLL4, INSM1, GCK, NR5A2
SLC25A25-AS1	0.484**	-1.930232	0.025533	ONECUT1, HNF1B, HNF4G, PDX1, NKX6-1, NEUROD1, GATA6, SOX9,, MNX1, RFX6, TCF7L2, TCF7, PROX1, GLIS3, PTF1A, CPA4, CHGA, CHGB, FEV,, DLL4, INSM1,, GCK, NR5A2
NNT-AS1	0.483**	-1.366922	0.007565	ONECUT1, HNF1B, HNF4G, PDX1, NKX6-1, NKX2-2, NEUROD1, GATA6, SOX9, MNX1, RFX6, TCF7L2, TCF7, PROX1, GLIS3, CPA4, CHGA, CHGB, FEV, PCSK1, DLL4, INSM1, GCK, NR5A2
LINC02381	0.481**	-2.495034	0.004835	ONECUT1, HNF1B, HNF4G, PDX1, NKX6-1, NKX2-2, NEUROD1, GATA6, SOX9, MNX1, RFX6, TCF7L2, TCF7, PROX1, GLIS3, CPA4, CHGA, CHGB, FEV, PCSK1, DLL4, INSM1, GCK, NR5A2
AC026471.1	0.481**	-1.16033	0.04654	ONECUT1, HNF1B, HNF4G, PDX1, NKX6-1, NKX2-2, NEUROD1, GATA6, SOX9, MNX1, RFX6, TCF7L2, TCF7, PROX1, GLIS3, CHGA, CHGB, PCSK1, DLL4, INSM1, NR5A2
MEG9	0.477**	-3.127263	0.000983	ONECUT1, HNF4G, PDX1,GATA4, TCF7L2, PROX1, GLIS3, PTF1A, PCSK1, DLL4,NR5A2
AP002748.4	0.475**	-1.18316	0.003262	ONECUT1, HNF1B, HNF4G, PDX1, NKX6-1,GATA6, SOX9, HES6, TCF7L2, TCF7, PROX1, GLIS3,NR5A2
AL021707.3	0.474**	-1.634677	0.000624	ONECUT1, HNF1B, HNF4G, PDX1, NKX6-1,GATA6, SOX9, MNX1,TCF7L2, TCF7, PROX1, GLIS3, CPA4, DLL4, NR5A2
AC018647.2	0.473**	-1.641918	0.02526	ONECUT1, HNF1B, HNF4G, PDX1, NKX6-1, NKX2-2, NEUROD1, GATA6, SOX9, MNX1, RFX6, TCF7L2, TCF7, PROX1, GLIS3, CHGA, CHGB, FEV, PCSK1, DLL4, INSM1, NR5A2
PRDM16-DT	0.470**	-2.579796	0.005967	ONECUT1, HNF1B, PDX1, NKX6-1, NKX2-2, NEUROD1, GATA6, SOX9, MNX1, RFX6, TCF7L2, PROX1, GLIS3, CPA4, CHGA, CHGB, FEV, DLL4, INSM1, GCK,
AL359921.2	0.468**	-1.675371	0.015848	ONECUT1, HNF1B, HNF4G, PDX1, NKX6-1, GATA6, SOX9, MNX1, TCF7L2, PROX1, GLIS3, DLL4, NR5A2
ELF3-AS1	0.462**	-1.453584	0.039806	ONECUT1, HNF1B, HNF4G, PDX1, NKX6-1, NKX2-2, GATA6, SOX9, HES6, MNX1, RFX6, TCF7L2, TCF7, PROX1, GLIS3, CPA4, CHGA, CHGB, FEV, PCSK1, DLL4, INSM1,NR5A2
H19	0.459**	-3.365472	0.004856	ONECUT1, HNF1B, HNF4G, PDX1, NKX6-1, GATA6, SOX9, MNX1, TCF7L2, TCF7, PROX1, GLIS3, PTF1A,DLL4, NR5A2
AC109460.2	0.457**	-1.344716	0.003872	ONECUT1, HNF1B, HNF4G, PDX1, NKX6-1, NKX2-2, NEUROD1, GATA6, SOX9, MNX1, RFX6, TCF7L2, TCF7, PROX1, GLIS3, CPA4, CHGB, PCSK1, DLL4, INSM1, NR5A2
AP000757.1	0.455**	-1.457171	0.002091	ONECUT1, HNF1B, PDX1, NKX6-1, NKX2-2, NEUROD1, GATA6, SOX9, MNX1, RFX6, TCF7L2, PROX1, GLIS3, CHGA, CHGB, FEV, PCSK1, INSM1, GCK
AC023509.3	0.453**	-1.542563	0.000571	ONECUT1, HNF1B, PDX1, NKX6-1, NKX2-2, NEUROD1, GATA6, SOX9, MNX1, RFX6, TCF7L2, PROX1, GLIS3, CHGA, CHGB, FEV, PCSK1, DLL4, INSM1
AL035661.1	0.452**	-2.278196	0.004812	ONECUT1, HNF1B, HNF4G, PDX1, GATA6, SOX9, MNX1, TCF7L2, PROX1, GLIS3, GP2, NR5A2
LINC00909	0.443**	-1.21341	0.009665	ONECUT1, HNF1B, HNF4G, PDX1, NKX6-1, NKX2-2, NEUROD1, GATA6, SOX9, MNX1, RFX6, TCF7L2, TCF7, PROX1, GLIS3, CHGA, CHGB, FEV, PCSK1, DLL4, INSM1, GCK, NR5A2
AC097468.3	0.441**	-1.98422	0.029327	ONECUT1, HNF1B, HNF4G, PDX1, NKX6-1, GATA6, SOX9, MNX1, RFX6, TCF7L2, PROX1, GLIS3, CPA4, DLL4, NR5A2
ZNF667-AS1	0.436**	-3.24304	6.2E-05	ONECUT1, HNF1B, HNF4G, PDX1, NKX6-1, NKX2-2, NEUROD1, GATA6, SOX9, MNX1, RFX6, TCF7L2, TCF7, PROX1, GLIS3, CHGA, CHGB, FEV, PCSK1, DLL4, INSM1, GCK, NR5A2
TMEM44-AS1	0.433**	-1.134186	0.003148	ONECUT1, HNF1B, HNF4G, PDX1, NKX6-1, NKX2-2, GATA6, SOX9, MNX1, TCF7L2, TCF7, PROX1, GLIS3, CHGB, FEV, INSM1, NR5A2
AL928654.2	0.431**	-1.305696	0.022488	ONECUT1, HNF1B, HNF4G, PDX1, NKX6-1, GATA6, SOX9, HES6, MNX1, TCF7L2, PROX1, GLIS3, CHGA, DLL4, NR5A2
EMSLR	0.424**	-1.536354	0.00847	ONECUT1, HNF1B, HNF4G, PDX1, GATA6, SOX9, HES6, MNX1, TCF7L2, TCF7, PROX1, GLIS3, PTF1A, CPA4, DLL4, NR5A2
MIR7-3HG	0.422**	-2.893704	0.014703	ONECUT1, HNF1B, HNF4G, PDX1, NKX6-1, NKX2-2, NEUROD1, SOX9, MNX1, RFX6, TCF7L2, PROX1, GLIS3, CHGA, CHGB, FEV, PCSK1, DLL4, INSM1, GCK, NR5A2
MNX1-AS1	0.421**	-2.081998	0.038035	HNF1B, PDX1, NKX6-1, NKX2-2, GATA6, SOX9,, MNX1, TCF7L2, PROX1, GLIS3, INSM1
AC108449.2	0.419**	-1.49049	0.030521	ONECUT1, HNF1B, HNF4G, PDX1, NKX6-1, NKX2-2, NEUROD1, GATA6, SOX9, RFX6, TCF7L2, TCF7, PROX1, GLIS3, CHGA, CHGB, PCSK1, DLL4, INSM1, NR5A2
NAPA-AS1	0.417**	-1.098776	0.027917	ONECUT1, HNF1B, HNF4G, PDX1, GATA6, SOX9, TCF7L2, TCF7, PROX1, GLIS3, GP2
LINC00957	0.414**	-1.202273	0.002339	ONECUT1, HNF1B, HNF4G, PDX1, NKX6-1, NKX2-2, NEUROD1, GATA6, SOX9, MNX1, RFX6, TCF7L2, PROX1, GLIS3, CPA4, CHGA, CHGB, FEV, DLL4, INSM1, GCK
CD2BP2-DT	0.412**	-1.367356	0.042524	HNF1B, HNF4G, PDX1, NKX6-1, GATA6, SOX9, TCF7L2, PROX1, GLIS3
AC004233.2	0.410**	-1.862911	0.017459	ONECUT1, HNF1B, HNF4G, PDX1, NKX6-1, NKX2-2, NEUROD1,, GATA6, SOX9, MNX1, RFX6, TCF7L2, PROX1, GLIS3, CPA4, CHGA, CHGB, FEV, PCSK1, DLL4, INSM1, GCK, NR5A2
LINC00342	0.409**	-1.382966	0.037571	ONECUT1, HNF1B, HNF4G, PDX1, NKX6-1,GATA6, SOX9,, MNX1, TCF7L2, TCF7, PROX1, GLIS3, CPA4, DLL4,GCK, NR5A2
AC005332.3	0.409**	-1.23033	0.003455	ONECUT1, HNF1B, HNF4G, PDX1, NKX6-1, NEUROD1, GATA6, SOX9, MNX1, RFX6, TCF7L2, TCF7, PROX1, GLIS3, CPA4, CHGA, CHGB, FEV, PCSK1, DLL4, INSM1, GCK, NR5A2
EXOC3-AS1	0.405**	-1.121631	0.009377	HNF1B, HNF4G, PDX1, NKX6-1, NKX2-2, GATA6, SOX9, MNX1, TCF7L2, PROX1, GLIS3, CHGB
AC092718.4	0.404**	-1.280606	0.005133	HNF1B, HNF4G, PDX1, NKX6-1, GATA6, SOX9, TCF7L2, TCF7, PROX1, GLIS3, PCSK1, DLL4, NR5A2
AL662797.2	0.402**	-2.409765	0.01791	HNF1B, HNF4G, PDX1, NKX6-1, GATA6, SOX9, MNX1, RFX6, TCF7L2, TCF7, PROX1, GLIS3, PCSK1, DLL4,, GCK, NR5A2
AC005046.2	0.401**	-1.533611	0.049076	ONECUT1, HNF1B, HNF4G, PDX1, NKX6-1, NKX2-2, NEUROD1, GATA6, SOX9, MNX1, RFX6, TCF7L2, PROX1, GLIS3, CHGA, CHGB, PCSK1, DLL4, NR5A2
AP000345.2	0.400**	-2.209009	0.000571	HNF1B, PDX1, NKX6-1, GATA6, SOX9, MNX1, TCF7L2, PROX1, GLIS3, CPA4, DLL4
ZNF32-AS2	0.398**	-1.612416	0.018084	ONECUT1, HNF1B, HNF4G, PDX1, NKX6-1, NKX2-2, NEUROD1,, GATA6, SOX9, MNX1, RFX6, TCF7L2, TCF7, PROX1, GLIS3, CPA4, CHGA, CHGB, FEV, PCSK1, DLL4, INSM1, NR5A2
GPRC5D-AS1	0.394**	-2.087789	0.000429	ONECUT1, HNF1B, HNF4G, PDX1, NKX6-1, NKX2-2, NEUROD1, GATA6, SOX9, MNX1, RFX6, TCF7L2, TCF7, PROX1, GLIS3, CHGA, CHGB, FEV, PCSK1, INSM1, NR5A2
SNHG14	0.393**	-1.783735	0.021069	ONECUT1, HNF1B, HNF4G, PDX1, NKX6-1, NKX2-2, NEUROD1, GATA6, SOX9, MNX1, RFX6, TCF7L2, TCF7, PROX1, GLIS3, CPA4, CHGA, CHGB, FEV, PCSK1, INSM1, GCK, NR5A2
AL158163.2	0.383**	-1.687435	0.003219	ONECUT1, HNF1B, HNF4G, PDX1, GATA6, SOX9, MNX1, TCF7L2, PROX1, GLIS3, GP2, NR5A2
AC090152.1	0.379**	-2.276363	0.003367	HNF1B, HNF4G, NKX2-2, PROX1, GLIS3, FEV
AC097639.1	0.377**	-2.647647	0.002051	ONECUT1, HNF1B, HNF4G, PDX1, NKX6-1,GATA6, SOX9, MNX1, TCF7L2,, PROX1, GLIS3, CHGB, NR5A2
U73166.1	0.373**	-1.429991	0.029312	ONECUT1, HNF1B, HNF4G, PDX1, NKX6-1, GATA6, SOX9, MNX1, TCF7L2, PROX1, GLIS3, DLL4
PSMG3-AS1	0.371**	-1.504912	0.004101	ONECUT1, HNF1B,, PDX1, NKX6-1, GATA6, SOX9, MNX1, TCF7L2, PROX1, GLIS3, INSM1
AL355102.4	0.371**	-1.429425	0.015528	ONECUT1, HNF1B, HNF4G, PDX1, NKX6-1, SOX9, HES6, MNX1, TCF7L2, PROX1, GLIS3, PTF1A, CPA4, DLL4, NR5A2
ATP6V0E2-AS1	0.370**	-1.797372	0.004396	HNF1B, PDX1, NKX6-1, GATA6, SOX9, MNX1, RFX6, TCF7L2, PROX1, GLIS3, FEV, DLL4, INSM1, GCK
AC092329.4	0.368**	-1.480944	0.00113	ONECUT1, HNF1B, HNF4G, PDX1, NKX6-1, GATA6, SOX9, MNX1, RFX6, TCF7L2, TCF7, PROX1, GLIS3, FEV, DLL4, GCK, NR5A2
MNX1-AS2	0.368**	-1.220973	0.016023	ONECUT1, HNF1B, HNF4G, PDX1, NKX6-1, GATA6, SOX9, HES6, MNX1, TCF7L2, PROX1, GLIS3, DLL4, GCK, NR5A2
LINC00511	0.365**	-2.227203	0.0021	ONECUT1, HNF1B, PDX1, NKX6-1, SOX9, MNX1, TCF7L2, PROX1, GLIS3, PTF1A, FEV, DLL4, GCK, NR5A2
TAPT1-AS1	0.365**	-1.344471	0.007551	ONECUT1, HNF1B, HNF4G, PDX1, NKX6-1, NEUROD1, GATA6, SOX9, RFX6, TCF7L2, PROX1, GLIS3, CPA4, DLL4, INSM1
SDCBP2-AS1	0.364**	-1.668813	0.012552	ONECUT1, HNF1B, HNF4G, PDX1, NKX6-1, NKX2-2, NEUROD1, GATA6, SOX9, MNX1, RFX6, TCF7L2, TCF7, PROX1, GLIS3, CHGB, FEV, PCSK1, INSM1, NR5A2
FAM111A-DT	0.356**	-1.744908	0.026671	ONECUT1, HNF1B, HNF4G, PDX1, NKX6-1, GATA6, SOX9, MNX1, RFX6, TCF7L2, TCF7, PROX1, GLIS3, CPA4, FEV, DLL4, GCK, NR5A2
AP000873.2	0.356**	-1.385981	0.006537	ONECUT1, HNF1B, HNF4G, PDX1, NKX6-1, GATA6, SOX9, MNX1, RFX6, TCF7L2, PROX1, GLIS3, CHGA, DLL4, NR5A2
ZNF790-AS1	0.353**	-1.229759	0.007888	ONECUT1, HNF1B, HNF4G, PDX1, NKX6-1, NEUROD1, GATA6, SOX9, MNX1, RFX6, TCF7L2, PROX1, GLIS3, CHGA, FEV, DLL4, INSM1, GCK, NR5A2
MCM3AP-AS1	0.348**	-1.023741	0.049512	ONECUT1, HNF1B, HNF4G, PDX1, NKX6-1, NEUROD1, GATA6, SOX9, MNX1, RFX6, TCF7L2, PROX1, GLIS3, CHGA, FEV, DLL4, INSM1, GCK, NR5A2
LINC00261	0.347**	-3.075824	0.016451	ONECUT1, PDX1, NKX6-1, GATA4, MNX1, TCF7L2, GLIS3
AC135050.5	0.347**	-1.609785	0.008387	ONECUT1, HNF1B, HNF4G, PDX1, NKX2-2, GATA6, SOX9, TCF7L2, PROX1, GLIS3, CHGB, PCSK1,GP2, NR5A2
AL022328.1	0.347**	-1.043931	0.012371	ONECUT1, HNF1B, HNF4G, PDX1, NKX6-1, NKX2-2, GATA6, SOX9, MNX1, RFX6, TCF7L2, PROX1, GLIS3, CPA4, DLL4, INSM1, NR5A2
AC004067.1	0.346**	-1.063812	0.01286	HNF1B, HNF4G, PDX1, NKX6-1, GATA6, SOX9, MNX1, TCF7L2, PROX1, GLIS3, DLL4, GCK
AP001107.4	0.344**	-1.928512	0.011238	ONECUT1, HNF1B, PDX1, NKX6-1, NKX2-2, NEUROD1, GATA6, SOX9, MNX1, RFX6, TCF7L2, PROX1, GLIS3, CHGA, CHGB, FEV, DLL4, INSM1, GCK,
AC004918.5	0.343**	-1.633831	0.002487	ONECUT1, HNF1B, HNF4G, PDX1, NKX6-1, NKX2-2, GATA6, SOX9, TCF7L2, TCF7, PROX1, GLIS3, CHGB, PCSK1, NR5A2
AL049775.1	0.341**	-1.430057	0.013752	ONECUT1, HNF1B, HNF4G, GATA6, SOX9, TCF7L2, PROX1, GLIS3, NR5A2
AL158163.1	0.339**	-1.03785	0.009069	HNF1B, HNF4G, PDX1, GATA6, MNX1, TCF7L2, PROX1, GLIS3, GP2, NR5A2
AC015849.3	0.336**	-1.60036	0.04537	ONECUT1, HNF1B, HNF4G, PDX1, NKX2-2, GATA6, SOX9, MNX1, RFX6, TCF7L2, PROX1, GLIS3, CHGB, NR5A2
AC091563.1	0.335**	-1.142587	0.002025	ONECUT1, HNF1B, HNF4G, PDX1, NKX6-1, NEUROD1, GATA6, SOX9, MNX1, RFX6, TCF7L2, PROX1, GLIS3, CHGA, FEV, PCSK1, DLL4, INSM1, GCK
AC107375.1	0.327**	-1.494579	0.046993	ONECUT1, HNF1B, HNF4G, PDX1, NKX6-1, NKX2-2, NEUROD1, GATA6, SOX9, MNX1, RFX6, TCF7L2, PROX1, GLIS3, CHGA, FEV, DLL4, INSM1, GCK, NR5A2
AL662844.4	0.325**	-1.250925	0.039572	ONECUT1, HNF1B, HNF4G, PDX1, NKX6-1, GATA6, SOX9, MNX1, RFX6, TCF7L2, PROX1, GLIS3, PTF1A, CPA4, FEV, DLL4, INSM1, GCK, NR5A2
AC011477.2	0.322**	-1.714697	0.016455	ONECUT1, HNF1B, HNF4G, PDX1, NKX6-1, GATA6, SOX9, MNX1, RFX6, TCF7L2, TCF7, PROX1, GLIS3, CHGB, FEV, DLL4, GCK, NR5A2
PINK1-AS	0.322**	-1.208215	0.02927	ONECUT1, HNF1B, HNF4G, PDX1, NKX6-1, GATA6, SOX9, RFX6, TCF7L2, TCF7, PROX1, GLIS3, CHGB, PCSK1, INSM1
LINC00174	0.321**	-1.342235	0.04753	ONECUT1, HNF1B, PDX1, NKX6-1, NKX2-2, NEUROD1, GATA6, SOX9, MNX1, RFX6, TCF7L2, PROX1, GLIS3, CPA4, DLL4, INSM1, GCK
LINC01963	0.318**	-2.455544	0.003181	HNF1B, HNF4G, PDX1, NKX6-1, NKX2-2, NEUROD1, GATA6, SOX9, TCF7L2, PROX1, GLIS3, CHGB, PCSK1, INSM1
AC011498.2	0.314**	-1.585168	0.00558	ONECUT1, HNF1B,, PDX1, NKX6-1, NKX2-2, NEUROD1,, GATA6, SOX9, MNX1, RFX6, TCF7L2, PROX1, GLIS3, CPA4, CHGA, CHGB, FEV, DLL4, INSM1, GCK
AC018521.4	0.311**	-3.32837	0.000104	ONECUT1, HNF4G, MNX1, TCF7L2, PROX1, GLIS3, CPA4, DLL4, NR5A2
ZNF32-AS1	0.311**	-1.049974	0.002135	ONECUT1, HNF1B, HNF4G, PDX1, NKX6-1, GATA6, SOX9, MNX1, RFX6, TCF7L2, PROX1, GLIS3, CPA4, CHGA, FEV, DLL4, INSM1, GCK, NR5A2
AC026801.2	0.310**	-1.162102	0.014154	HNF1B, PDX1, NKX6-1, NEUROD1, GATA6, SOX9, MNX1, RFX6, TCF7L2, PROX1, GLIS3, FEV, PCSK1, INSM1, GCK
AC093620.1	.305**	-1.180784	0.03222	PDX1, MNX1, TCF7L2, PROX1, GLIS3, DLL4, GCK
AL031717.1	0.301**	-1.589415	0.001643	ONECUT1, HNF1B, PDX1, NKX6-1, GATA6, SOX9, MNX1, TCF7L2, PROX1, GLIS3, DLL4
AL512791.1	0.300**	-1.628931	0.008489	ONECUT1, HNF1B, HNF4G, PDX1, NKX6-1, NKX2-2, GATA6, SOX9, MNX1, RFX6, TCF7L2, PROX1, GLIS3, CHGB, NR5A2
AC106782.5	0.300**	-1.011157	0.015448	ONECUT1, HNF1B, HNF4G, PDX1, NKX6-1, GATA6, SOX9, MNX1, RFX6, TCF7L2, TCF7, PROX1, GLIS3, CPA4, CHGA, DLL4, GCK, NR5A2
**Upregulated**				
AC104958.2	> 0.300**	3.06691	0.01042	ABCA1, GCKR, SLC3A2
AC008264.2	> 0.300**	2.64584	0.008036	ABCC2
DBH-AS1	> 0.300**	2.6249	0.033449	ABCA7
AC005261.4	> 0.300**	2.52597	0.002745	ABCA7, ABCC2, ANXA1, APOC1, BMP2, GCGR, GCKR, HKDC1, SLC16A1, SLC2A3, SLC3A2
AC092535.5	> 0.300**	2.46391	0.003268	ABCA7, GCGR
AC025857.2	> 0.300**	2.41229	0.006863	ABCA7, ANXA1, APOC1, APOC2, BMP2, GCKR, HKDC1, SLC16A1, SLC2A3
AP001453.4	> 0.300**	1.90867	0.00027	GCGR, GCKR
AC090510.3	> 0.300**	1.75372	0.002414	ABCA7, ABCC2, GCGR, GCKR, HKDC1, SLC16A1, SLC2A3
AC084036.1	> 0.300**	1.73696	0.045269	CEBPA
AC103706.1	> 0.300**	1.60426	0.0041	ABCA7, ABCC2, GCGR, GCKR, HKDC1, SLC16A1, SLC2A3, SLC3A2
ITGB2-AS1	> 0.300**	1.48263	0.005362	ANXA1, APOC1, APOC2, BMP2, GCKR, HKDC1, SLC16A1, SLC2A3
LINC01137	> 0.300**	1.43804	0.014543	ABCA1, ANXA1, APOC1, BMP2, CEBPA, GCGR, GCKR, HKDC1, SLC16A1, SLC3A2
AL161669.3	> 0.300**	1.43659	0.003279	ABCC2, GCGR, GCKR, SLC16A1, SLC2A3
AL390728.6	> 0.300**	1.42115	0.001923	ABCA1, ABCA7, ABCC2, ANXA1, BMP2, GCGR, GCKR, HKDC1, SLC16A1, SLC2A3, SLC3A2, WNT5A
AC009407.1	> 0.300**	1.373	0.018734	ABCC2, SLC2A2
AC111000.4	> 0.300**	1.3692	0.043736	ABCC2
AL161668.4	> 0.300**	1.35769	0.003348	ABCC2
AC005261.2	> 0.300**	1.32648	0.015732	ABCA7, ABCC2, ANXA1, BMP2, GCGR, GCKR, HKDC1, SLC16A1, SLC2A3, SLC3A2
CPNE8-AS1	> 0.300**	1.31393	0.033738	GCGR, GCKR
AL731533.2	> 0.300**	1.31069	0.000922	ABCC2, GCGR, SLC16A1
AC106739.1	> 0.300**	1.274	0.007299	GCKR
AL135924.2	> 0.300**	1.25907	0.018394	ABCC2
AC009690.2	> 0.300**	1.2405	0.031511	ABCA7, ABCC2, ANXA1, APOC1, BMP2, GCGR, GCKR, HKDC1, SLC16A1, SLC2A3
GAS6-DT	> 0.300**	1.15488	0.00105	ABCA1, ABCC2, ANXA1, GCGR, GCKR, HKDC1, SLC16A1, SLC2A3
AL080317.2	> 0.300**	1.15242	0.012166	ABCA7, ABCC2, GCGR
AC022144.1	> 0.300**	1.11867	0.047965	ABCA7, ABCC2, GCGR, GCKR, HKDC1, SLC16A1, SLC2A3, SLC3A2
AP001033.4	> 0.300**	1.09916	0.048671	ABCA7, BMP2, GCGR, GCKR, HKDC1, SLC16A1, SLC2A3, SLC3A2
LNCSRLR	> 0.300**	1.0583	0.00857	GCKR
RUSC1-AS1	> 0.300**	1.0481	0.002152	ABCA7, ABCC2, ANXA1, BMP2, GCGR, GCKR, HKDC1, SLC16A1, SLC2A3, SLC3A2

^**^indicates a highly significant correlation

The network analysis of the upregulated DE-lncRNAs was conducted by examining their correlation with selected upregulated genes identified in *FOXA2*^*−/−*^ PPs. These DEGs include *APOC2*, *GCGR*, *HKDC1*, *SLC2A3*, *ABCC2*, *SLC2A2*, *APOC1*, *CEBPA*, *GCKR*, *ANXA1*, *WNT5A*, *ABCA1*, *BMP2*, *SLC16A1*, *ABCA7*, and *SLC3A2* (Supplementary Table 3). Incorporating these results with lncRNA and mRNA expression data from 305 pancreatic tissue database revealed that only a limited number of upregulated DE-lncRNAs exhibited a strong correlation with the selected upregulated genes, as depicted in the constructed network (Supplementary Fig. 4 and Table 1). Among the correlated lncRNAs, those demonstrating the highest levels of upregulation included *AC104958.2*, *AC008264.2*, *DBH-AS1*, *AC005261.4*, *AC092535.5*, and *AC025857.2*.

### Characterization of lncRNA profiles in FOXA2 knockout iPSC-derived pancreatic islets

**Fig. 3:**
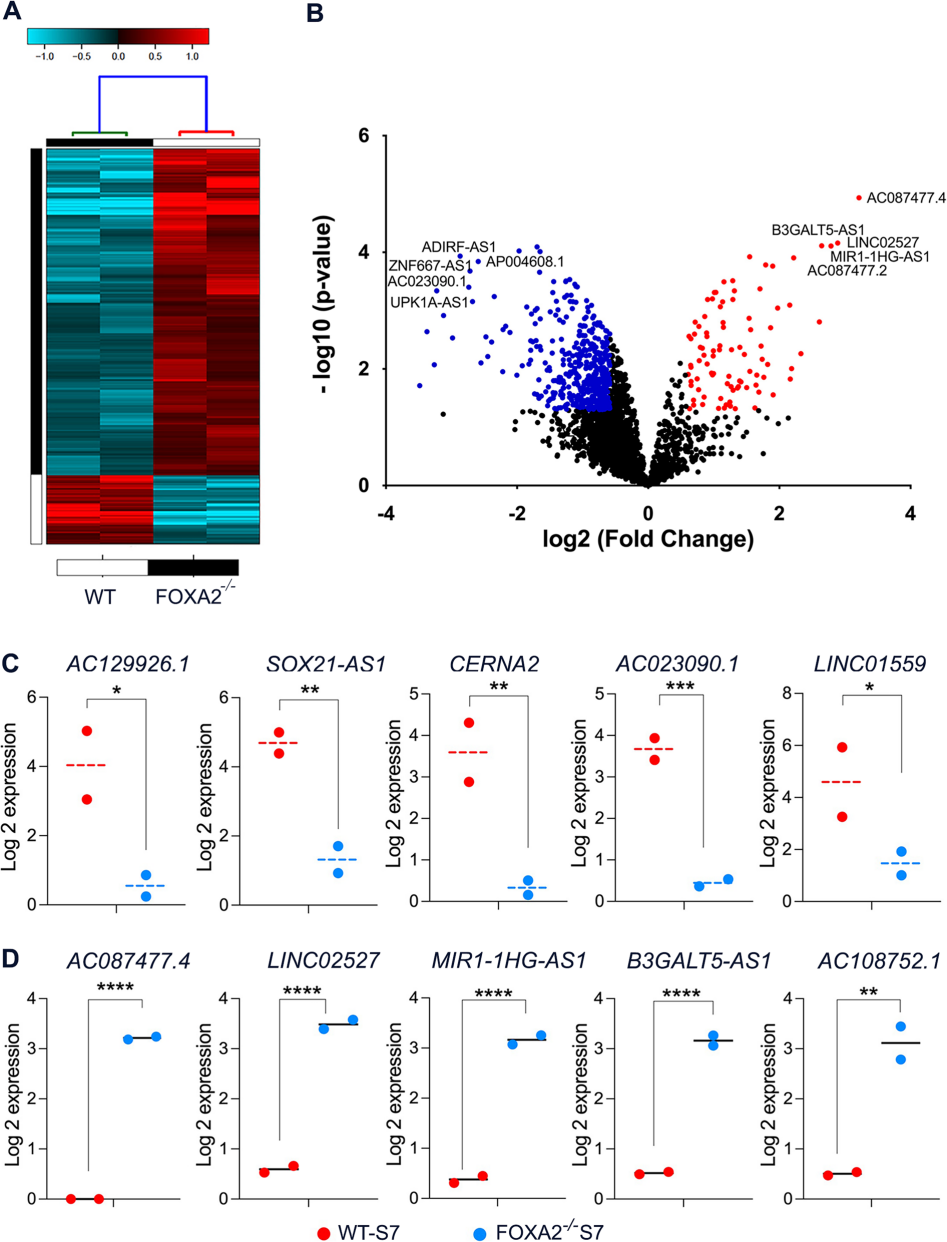
Differentially expressed lncRNAs in pancreatic islets derived from *FOXA2*^*−/−*^ iPSCs compared to those derived from WT-iPSCs. **A** Hierarchical clustering of captured lncRNAs in the pancreatic islets derived from *FOXA2*^*−/−*^ iPSCs and WT-iPSCs. Each column represents one differentiation experiment, and each row represents the lncRNA transcript. The expression level of each transcript (log2) is depicted according to the color scale. **B** Volcano plot depicting upregulated (red) and downregulated (blue) lncRNAs (*p* < 0.05, Log2 FC > 1). Graphs represent the expression of the top 5 downregulated (**C**) and upregulated (**D**) lncRNA transcripts

In order to investigate the impact of FOXA2 deletion on the expression of lncRNAs in pancreatic islets, we extracted the DE-lncRNAs and DEGs from RNA-seq data. This data was obtained by comparing the pancreatic islets derived from *FOXA2*^*–/–*^iPSCs and WT-iPSCs. Our transcriptomic analysis revealed a total of 1031 significantly downregulated DEGs (Log2 FC < -1.0, *p* < 0.05), and 734 significantly upregulated DEGs (Log2 FC > 1.0, *p* < 0.05) in pancreatic islets derived from *FOXA2*^*–/–*^ iPSCs compared to the WT controls. The relevant DEGs selected for the correlation study are listed in Supplementary Table 3. Furthermore, our RNA-Seq data revealed 517 DE-lncRNAs in *FOXA2*^*–/–*^pancreatic islets compared to WT-pancreatic islets. Among those DE-lncRNAs, 177 were significantly downregulated (Log2 FC <  − 1.0, *p* < 0.05) and 59 were significantly upregulated (Log2 FC > 1.0, *p* < 0.05) (Fig. 3A, B and Supplementary Table 5). The expression of the top 5 downregulated and top 5 upregulated DE-lncRNA transcripts in pancreatic islets derived from *FOXA2*^*–/–*^ iPSCs compared to the WT controls are shown in Fig. 3C and D. *AC129926.1* was the top downregulated and *AC087477.4* was the top upregulated DE-lncRNA in *FOXA2*^*–/–*^ pancreatic islets compared to WT-pancreatic islets.

### Correlations between DE-lncRNAs and DE-mRNAs in iPSC-derived pancreatic islets

**Fig. 4:**
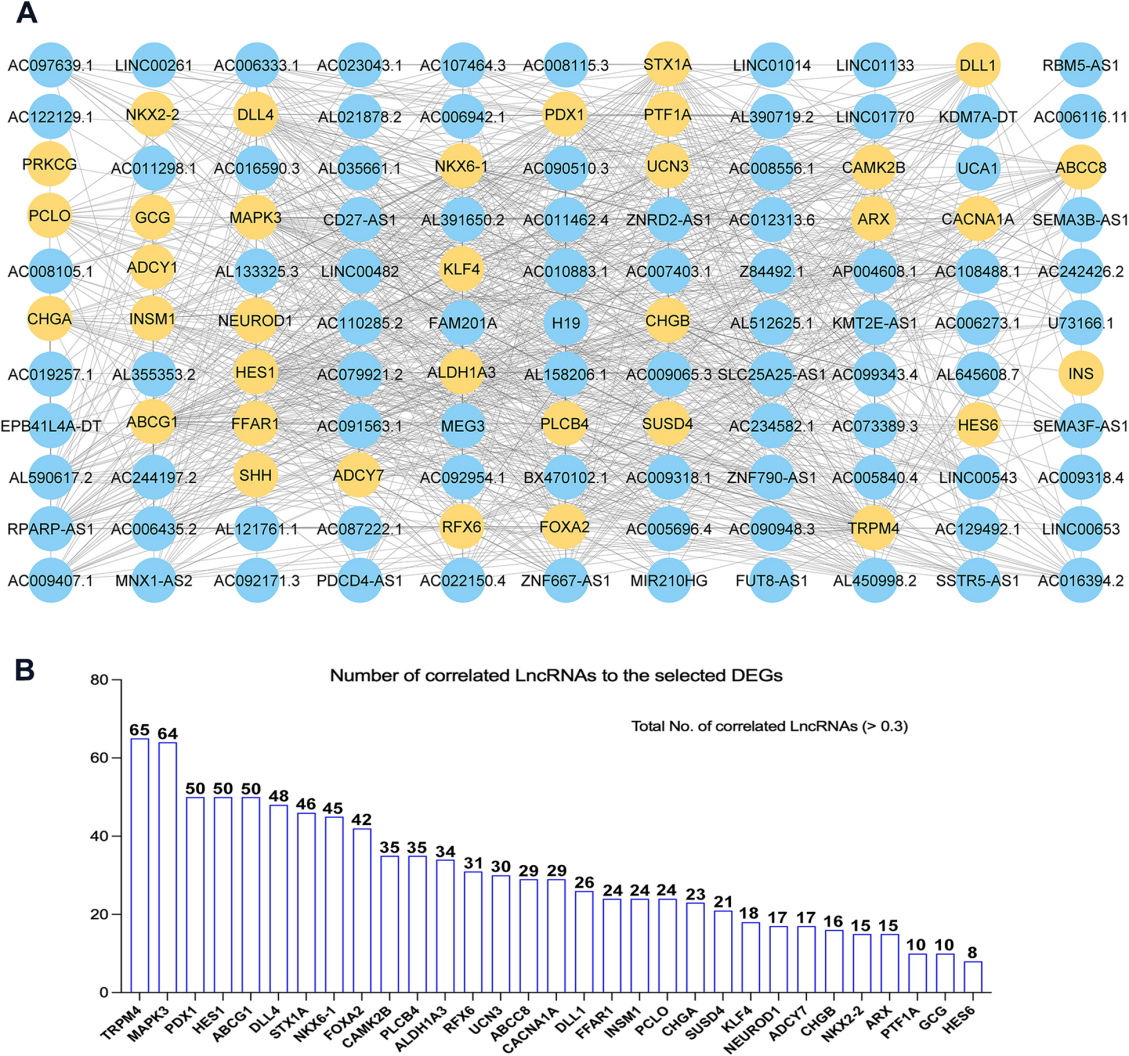
Co-expression network analysis of downregulated lncRNAs and DEGs in pancreatic islets derived from *FOXA2*.^*−/−*^iPSCs. The correlation analysis between the downregulated DE-lncRNAs and DEGs in the pancreatic islets (**A**). The number of lncRNAs correlated with each specific DEG is presented in graph (**B**)

To identify potential interaction, we constructed a co-expression network through correlation analysis between the DE-lncRNAs and key DE-mRNAs in iPSC-derived pancreatic islets. Our transcriptome analysis showed that mRNA expression of several pancreatic genes related to the development and function of pancreatic islet cells was significantly downregulated in iPSC-derived pancreatic islets lacking FOXA2 compared to WT controls (Supplementary Table 3). We selected key downregulated DE-mRNAs that are known to play an essential role in β-cell development and function, including *FOXA2*, *TRPM4*, *MAPK3*, *PDX1*, *HES*, *ABCG1*, *DLL4*, *STX1A*, *NKX6-1*, *PLCB4*, *CAMK2B*, *ALDH1A3*, *RFX6*, *UCN3*, *CACNA1A*, *ABCC8*, *DLL1*, *PCLO*, *INSM1*, *FFAR1*, *CHGA*, *SUSD4*, *KLF4*, *NEUROD1*, *ADCY7*, *CHGB*, *NKX2-2*, *ARX*, *PTF1A*, *GCG*, *HES6*, *SHH*, *PRKCG*, *INS*, *ADCY1*, *PPY*, and *IAPP.* We subsequently performed correlation analysis on those identified downregulated DE-lncRNAs and downregulated DE-mRNAs in normal pancreatic cells from GTEx data as well as in iPSC-derived pancreatic islets. Interactions with Pearson correlation coefficients > 0.3 were selected and the co-expression network analysis was constructed (Fig. 4A; Supplementary Fig. 5). The analysis revealed 86 downregulated DE-lncRNAs with a strong correlation (> 0.3) to the selected downregulated DE-mRNAs. Out of these DE-lncRNAs, 42 DE-lncRNAs showed a strong correlation with FOXA2, listed with its correlated mRNAs in Table 2. The commonly correlated downregulated DE-lncRNAs with FOXA2 and other DEGs are shown in Fig. 4B and Table 2. The analysis revealed that *ZNF667-AS1*, *AL035661.1*, *AL390719.2*, *AC091563.1*, *AC090510.3*, *MEG3*, *LINC00261*, *U73166.1*, *AC097639.1*, *MNX1-AS2,* and *H19* were the top downregulated DE-lncRNAs with a strong correlation to *FOXA2* (Supplementary Fig. 6). There were 65, 64, 50, 50, 50, 48, 46, and 45, DE-lncRNAs correlated with *TRPM4*, *MAPK3*, *PDX1, HES6*, *ABCG1, DLL4*, *STX1A*, and *NKX6.1* (Fig. 4B). As previously known, FOXA2 commonly regulates PDX1, NKX6.1, NKX2.2 and ABCC8 TFs. We identified 23 lncRNAs that are commonly and strongly correlated to *FOXA2*, *PDX1*, *NKX6.1* and *ABCC8,* with the lowest log2 fold expression for *ZNF667-AS1*, *AL390719.2*, *AC091563.1*, *AC090510.3*, and *AC097639.1,* as shown in Fig. 4C and Supplementary Fig. 6 and Supplementary Table 6.

**Table 2:** List of the strongly correlated lncRNAs to FOXA2 with Pearson correlation (PC) > 0.3 in the pancreatic islets derived from *FOXA2*^*−/−*^iPSCs compared with those derived from WT-iPSCs (*p* < 0.05)

lncRNA	PC	Log2-FC	*P*-value	Other TFs correlated to the lncRNA
**Downregulated**				
LINC00543	0.636**	-1.2969645	0.00158635	ABCG1, ALDH1A3,CHGA,CHGB, GCG, HES1, HES6, KLF4, MAPK3, NKX2-2, NKX6-1, PDX1, STX1A, SUSD4, TRPM4
AL390719.2	0.587**	-1.9211931	0.00881401	ABCC8, ABCG1, ALDH1A3, CHGB, DLL1, DLL4, FFAR1, HES1, HES6, KLF4, MAPK3, NKX2-2, NKX6-1, PDX1, STX1A, SUSD4, TRPM4,UCN3
AC008556.1	0.546**	-1.2948708	0.00696662	ALDH1A3, DLL4, HES1, HES6, MAPK3, PDX1, PTF1A, TRPM4
MEG3	0.518**	-1.6955374	8.0682E-05	ABCG1, ALDH1A3, DLL1, DLL4, FFAR1, HES1, INSM1, MAPK3, NKX6-1, PDX1, PTF1A, RFX6, STX1A, TRPM4, UCN3
AC006333.1	0.517**	-1.0062851	0.03861764	ABCC8, ABCG1, ALDH1A3, CACNA1A, CAMK2B, CHGA, CHGB, DLL1, DLL4, FFAR1, GCG, HES1, INSM1, KLF4, MAPK3, NEUROD1, NKX2-2, NKX6-1, PCLO, PDX1, PLCB4, RFX6, STX1A, SUSD4, TRPM4, UCN3
RPARP-AS1	0.497**	-1.1768327	0.00054477	ABCC8, ABCG1, ALDH1A3, ARX, CACNA1A, CAMK2B, CHGA, CHGB, DLL1, DLL4, FFAR1, GCG, HES1, HES6, INSM1, KLF4, MAPK3, NEUROD1, NKX2-2, NKX6-1, PDX1, PLCB4, RFX6, STX1A, SUSD4, TRPM4, UCN3
SLC25A25-AS1	0.484**	-1.0534922	0.00894033	ABCC8, ABCG1, ALDH1A3, CACNA1A, CAMK2B, CHGA, CHGB, DLL1, DLL4, FFAR1, HES1, INSM1, MAPK3, NEUROD1, NKX6-1, PDX1, PLCB4, PTF1A, RFX6, STX1A, TRPM4, UCN3
PDCD4-AS1	0.484**	-1.1102287	0.00770037	ABCG1, ALDH1A3, DLL1, DLL4, HES1, KLF4, MAPK3, PDX1, STX1A, SUSD4, TRPM4
CD27-AS1	0.470**	-1.1406858	0.02874059	ABCC8, ABCG1, ADCY7, ALDH1A3, CACNA1A, CAMK2B, CHGA, CHGB, DLL1, DLL4, FFAR1, HES1, INSM1, KLF4, MAPK3, NEUROD1, NKX2-2, NKX6-1, PCLO, PDX1, PLCB4, RFX6, STX1A, SUSD4, TRPM4, UCN3
AC108488.1	0.466**	-1.0384066	0.04593561	ABCC8, ABCG1, ALDH1A3, CACNA1A, CAMK2B, CHGA, DLL1, DLL4, FFAR1, HES1, INSM1, KLF4, MAPK3, NEUROD1, NKX2-2, NKX6-1, PDX1, PLCB4, RFX6, STX1A, SUSD4, TRPM4, UCN3
LINC01770	0.463**	-1.4111367	0.00119341	ABCG1, ADCY7, ALDH1A3, DLL1, DLL4, HES1, MAPK3, NKX6-1, PDX1, PTF1A, STX1A, TRPM4, UCN3
H19	0.459**	-1.0508881	0.00682397	ABCG1, ALDH1A3, DLL1, DLL4, HES1, MAPK3, NKX6-1, PDX1, PTF1A, TRPM4
AL035661.1	0.452**	-2.1081528	0.00237583	FOXA2, ALDH1A3, KLF4, MAPK3, PDX1, SUSD4
AC099343.4	0.443**	-1.0656839	0.02841831	ABCG1, ADCY7, ALDH1A3, CACNA1A, CHGA, DLL1, DLL4, FFAR1, HES1, KLF4, MAPK3, NKX6-1, PCLO, PDX1, PLCB4, PTF1A, RFX6, STX1A, TRPM4, UCN3
ZNF667-AS1	0.436**	-2.7175609	0.00021026	ABCC8, ABCG1, ADCY7, ALDH1A3, ARX, CACNA1A, CAMK2B, CHGA, CHGB, DLL1, DLL4, FFAR1, GCG, HES1, INSM1, MAPK3, NEUROD1, NKX2-2, NKX6-1, PCLO, PDX1, PLCB4, RFX6, STX1A, SUSD4, TRPM4, UCN3
AL158206.1	0.434**	-1.2478764	0.041127	ABCG1, ALDH1A3, DLL1, DLL4, HES6, KLF4, MAPK3, PDX1, STX1A, SUSD4, TRPM4
SEMA3B-AS1	0.434**	-1.2944753	0.00843693	ALDH1A3, DLL1, HES1, KLF4, MAPK3, PDX1, SUSD4, TRPM4
ZNRD2-AS1	0.424**	-1.0543007	0.00266566	ABCC8, ABCG1, ADCY7, ALDH1A3, CACNA1A, CAMK2B, CHGA, CHGB, DLL1, DLL4, FFAR1, GCG, HES1, INSM1, MAPK3, NEUROD1, NKX6-1, PDX1, PTF1A, RFX6, STX1A, TRPM4, UCN3
LINC00482	0.414**	-1.0932474	0.00573003	ABCG1, ALDH1A3, CACNA1A, CAMK2B, DLL1, DLL4, HES1, MAPK3, NKX6-1, PDX1, PLCB4, PTF1A, STX1A, TRPM4, UCN3
AL450998.2	0.413**	-1.0090053	0.00579397	ABCC8, ABCG1, ADCY7, ALDH1A3, CACNA1A, CAMK2B, CHGA, CHGB, DLL4, FFAR1, GCG, HES1, INSM1, MAPK3, NEUROD1, NKX2-2, NKX6-1, PDX1, PLCB4, RFX6, STX1A, SUSD4, TRPM4, UCN3
AC234582.1	0.397**	-1.0557378	0.01377192	ABCC8, ABCG1, ADCY7, ALDH1A3, CACNA1A, CAMK2B, CHGA, DLL4, HES1, MAPK3, NKX6-1, PDX1, RFX6, STX1A, TRPM4, UCN3
KDM7A-DT	0.394**	-1.0891695	0.03269201	ABCG1, ALDH1A3, ARX, CAMK2B, HES1, INSM1, KLF4, MAPK3, NKX6-1, PCLO, PDX1, PLCB4, RFX6, STX1A, TRPM4, UCN3
AC006942.1	0.383**	-1.1682946	0.03467244	MAPK3, NKX6-1, PDX1, TRPM4
FAM201A	0.377**	-1.0741873	0.00589022	ABCC8, ABCG1, ADCY7, ALDH1A3, CAMK2B, DLL4, HES1, MAPK3, NKX6-1, PDX1, RFX6, STX1A, SUSD4, TRPM4, UCN3
AC097639.1	0.377**	-1.5418811	0.01047655	ABCC8, ABCG1, ALDH1A3, CHGB, DLL1, KLF4, MAPK3, NKX6-1, PDX1, SUSD4, TRPM4
U73166.1	0.373**	-1.5707679	0.00789093	ABCG1, ALDH1A3, DLL1, DLL4, HES1, MAPK3, NKX6-1, PDX1, STX1A, SUSD4, TRPM4
SEMA3F-AS1	0.372**	-1.0500171	0.0079954	ABCG1, DLL1, DLL4, HES1, KLF4, MAPK3, PDX1, STX1A, SUSD4, TRPM4
MNX1-AS2	0.368**	-1.1191168	0.01121461	ABCC8, ABCG1, CACNA1A, CAMK2B, DLL1, DLL4, FFAR1, HES1, HES6, MAPK3, NKX6-1, PDX1, STX1A, TRPM4
KMT2E-AS1	0.361**	-1.0672028	0.01315702	ABCC8, ABCG1, CAMK2B, CHGA, DLL4, FFAR1, HES1, MAPK3, NKX6-1, PDX1, PTF1A, STX1A, TRPM4, UCN3
AC011462.4	0.355**	-1.4430689	0.00031872	ABCC8, ABCG1, ALDH1A3, CAMK2B, DLL1, DLL4, FFAR1, HES1, MAPK3, NKX6-1, PDX1, PTF1A, RFX6, STX1A, TRPM4, UCN3
ZNF790-AS1	0.353**	-1.3528114	0.00145091	ABCC8, ABCG1, ALDH1A3, ARX, CACNA1A, CAMK2B, CHGA, DLL1, DLL4, FFAR1, HES1, INSM1, KLF4, MAPK3, NEUROD1, NKX6-1, PCLO, PDX1, PLCB4, RFX6, STX1A, TRPM4, UCN3
AC006435.2	0.350**	-1.2727405	0.04433202	ABCC8, ABCG1, ADCY7, ALDH1A3, CACNA1A, CAMK2B, CHGA, CHGB, DLL1, DLL4, FFAR1, GCG, HES1, INSM1, MAPK3, NEUROD1, NKX2-2, NKX6-1, PCLO, PDX1, PLCB4, RFX6, STX1A, TRPM4, UCN3
LINC00261	0.347**	-1.6508816	9.7549E-05	CACNA1A, NKX6-1, PDX1, PLCB4
AC010883.1	0.335**	-1.3060496	0.03125186	ABCG1, ALDH1A3, CAMK2B, DLL4, HES1, MAPK3, NKX6-1, PDX1, PLCB4, STX1A, TRPM4
AC091563.1	0.335**	-1.8529012	0.00086475	ABCC8, ABCG1, ADCY1, ALDH1A3, ARX, CACNA1A, CAMK2B, CHGA, DLL4, FFAR1, HES1, INSM1, MAPK3, NEUROD1, NKX6-1, PCLO, PDX1, PLCB4, RFX6, STX1A, TRPM4, UCN3
AL590617.2	0.322**	-1.1898488	0.00069819	ABCC8, ABCG1, ADCY7, CACNA1A, CAMK2B, CHGA, DLL4, FFAR1, GCG, HES1, HES6, INSM1, MAPK3, NEUROD1, NKX2-2, NKX6-1, PCLO, PDX1, PLCB4, RFX6, STX1A, TRPM4, UCN3
AC090510.3	0.319**	-1.7243884	0.00167289	ABCC8, ABCG1, CACNA1A, CAMK2B, CHGA, DLL1, DLL4, FFAR1, HES1, INSM1, MAPK3, NKX6-1, PCLO, PDX1, PLCB4, RFX6, STX1A, TRPM4, UCN3
AC110285.2	0.313**	-1.5091246	0.00943956	ABCC8, KLF4, MAPK3, NKX6-1, PDX1, STX1A, SUSD4, TRPM4
MIR210HG	0.306**	-1.3568307	0.0139073	ABCC8, ABCG1, ALDH1A3, ARX, CACNA1A, CAMK2B, CHGA, DLL1, DLL4, FFAR1, HES1, INSM1, KLF4, MAPK3, NEUROD1, NKX6-1, PCLO, PDX1, PLCB4, RFX6, STX1A, TRPM4, UCN3
AC016394.2	0.305**	-1.4490727	0.03173317	ABCC8, ABCG1, CACNA1A, CAMK2B, CHGA, DLL4, FFAR1, HES1, INSM1, MAPK3, NKX6-1, PDX1, PLCB4, RFX6, STX1A, TRPM4, UCN3
AC005840.4	0.303**	-1.2066464	0.00699161	ABCG1, ALDH1A3, DLL1, DLL4, HES6, KLF4, MAPK3, PDX1, STX1A, SUSD4, TRPM4
AC107464.3	0.303**	-1.4874602	0.03289592	ABCC8, ABCG1, ARX, CACNA1A, CAMK2B, DLL4, HES1, MAPK3, NKX6-1, PCLO, PDX1, PLCB4, RFX6, STX1A, TRPM4, UCN3
**Upregulated**				
B3GALT5-AS1	> 0.30**	2.64437	7.75E-05	ATP1B1, ATP1B2, FXYD2, SST, HHEX, ABCC2, SOX9, BMP4, WNT6, MYC
AC087477.2	> 0.30**	2.21789	0.000124	WNT6, LEF1
AC027031.2	> 0.30**	1.90392	0.027906	CPA4
DNM3OS	> 0.30**	1.89734	0.000172	ATP1B1, ATP1B2, FXYD2, PLA2G2A, KCNQ1, HHEX, SOX9, BMP4, DPP4
AC124067.4	> 0.30**	1.79024	0.000165	WNT6, LEF1
GATA2-AS1	> 0.30**	1.72026	0.004055	ATP1B1, CPA4, PLA2G2A, KCNQ1, HHEX, SOX9
AC111000.4	> 0.30**	1.7032	0.000423	ABCC2
SPATA8	> 0.30**	1.54558	0.00012	ABCC2
AL049775.1	> 0.30**	1.27009	0.004693	KCNQ1, HHEX, SOX9
LINC00648	> 0.30**	1.16137	0.046814	ABCC2, WNT6
AC080038.2	> 0.30**	1.10511	0.032125	ATP1B1, ATP1B2, FXYD2, SST, HHEX, BMP4
HOXA-AS2	> 0.30**	1.03006	0.000488	ABCC2, WNT6

^**^indicates a highly significant correlation

Similarly, we conducted a correlation analysis to establish connections between the upregulated DE-lncRNAs to the selected upregulated DEGs identified in *FOXA2*^*−/−*^ pancreatic islets. These DEGs included *ATP1B1*, *ATP1B2*, *FXYD2*, *CPA4*, *PLA2G2A*, *KCNQ1*, *SST*, *HHEX*, *ABCC2*, *BMP4*, *WNT6*, and *MYC* (Supplementary Table 3). Their incorporation with the database of 305 pancreatic tissue profiles resulted in identification of 12 upregulated DE-lncRNAs with Pearson correlation coefficients > 0.3, as enlisted in Table 2. Among them, the highly upregulated lncRNAs were *B3GALT5-AS1*, *AC087477.2*, *AC027031.2*, *DNM3OS*, and *AC124067.4* (Supplementary Fig. 7 and Table 2). Using the identical analysis approach on our RNA-Seq data, we observed that the upregulated DE-mRNAs exhibited associations with the same DE-lncRNAs identified in pancreatic tissues (Supplementary Fig. 8).

### Identifications of commonly DE-lncRNAs in pancreatic progenitors and islets derived from FOXA2 ^***−/−***^ iPSCs

**Fig. 5:**
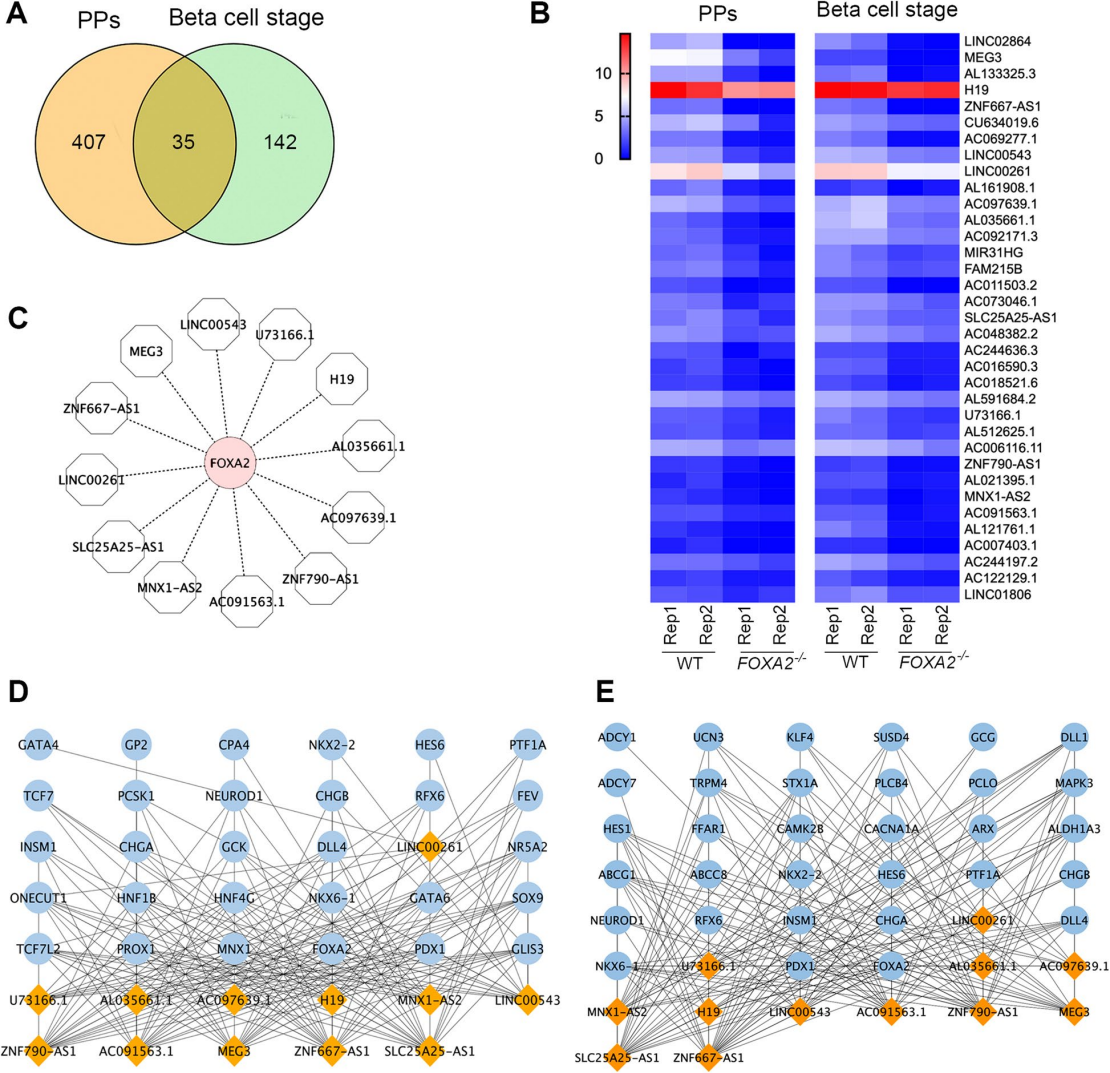
Identification of commonly downregulated lncRNAs in pancreatic progenitors and islets. **A** A Venn diagram showing the number of lncRNAs that are downregulated in pancreatic progenitor (PPs) and pancreatic islets stages. **B** Heatmaps showing the expression of the commonly downregulated lncRNAs in PPs and pancreatic islets stages. **C** The commonly downregulated lncRNAs in both stages showed a strong correlation with FOXA2. Constructed networks to explore the global correlation of these FOXA2-strongly correlated lncRNAs with other DEGs in PPs (**D**) and pancreatic islet stage (**E**)

**Fig. 6:**
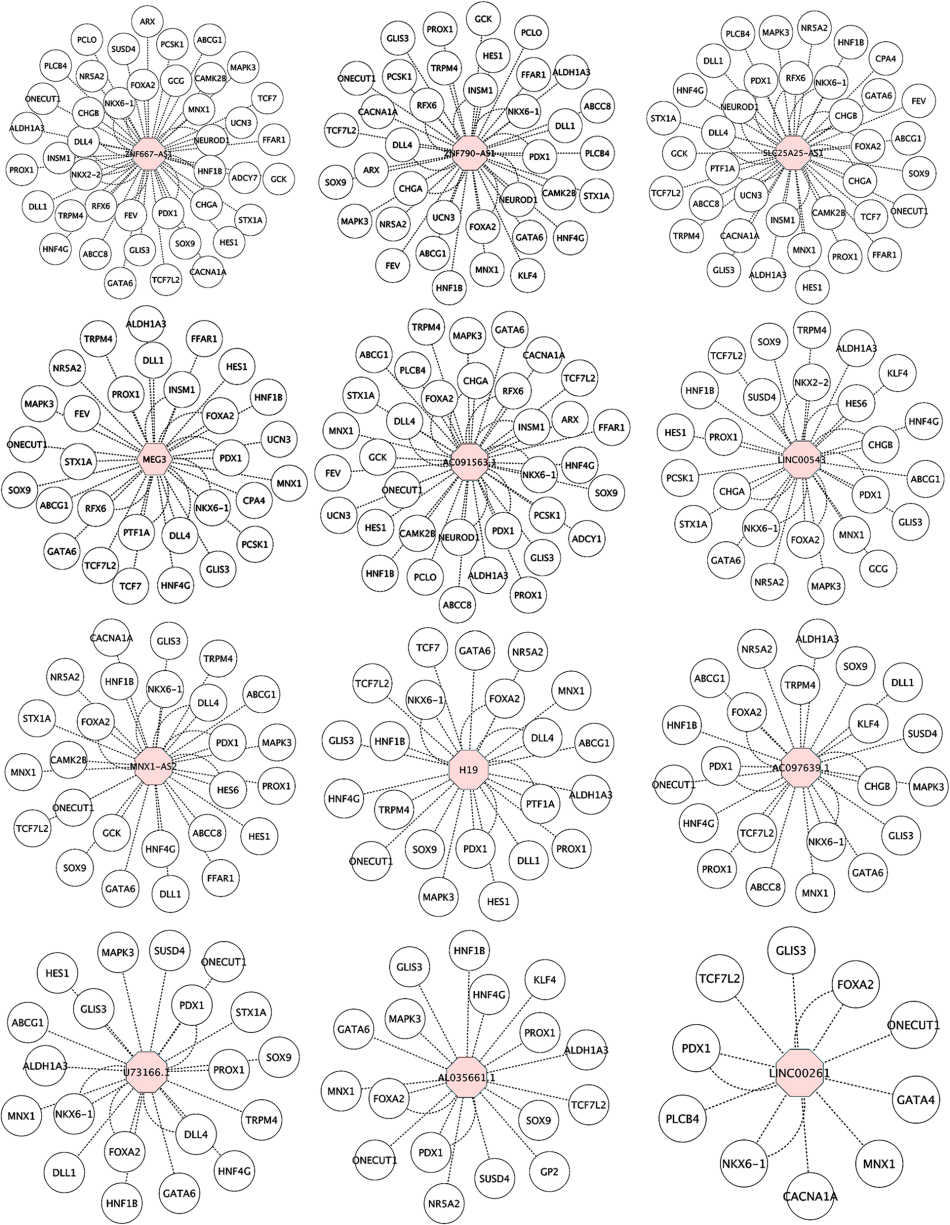
Individual networks for each commonly downregulated lncRNA exhibited strong correlation to FOXA2. The networks were constructed for *MEG3*, *H19*, *ZNF667-AS1*, *LINC00543*, *LINC00261*, *AC097639.1*, *AL035661.1*, *SLC25A25-AS1*, *U73166.1*, *ZNF790-AS1*, *MNX1-AS2*, and *AC091563.1*. The curved lines represent the transcription factors, which are commonly correlated to the lncRNA in both stages of PPs and pancreatic islets

Next, we aimed to identify commonly dysregulated lncRNAs in both PPs and pancreatic islets. We identified 35 lncRNAs that were downregulated in PPs and pancreatic islets lacking FOXA2 compared to WT controls (Fig. 5A, B; Table 3). Of those downregulated DE-lncRNAs, only 12 lncRNAs had a strong Pearson correlation > 0.3 with FOXA2 including *MEG3*, *H19*, *ZNF667-AS1*, *LINC00543*, *LINC00261*, *AC097639.1*, *AL035661.1*, *SLC25A25-AS1*, *U73166.1*, *ZNF790-AS1*, *MNX1-AS2*, and *AC091563.1* (Fig. 5C). Furthermore, we showed the global correlation between these 12 lncRNAs and the specific mRNAs in the PPs and pancreatic islets in normal pancreatic cells from GTEx data (Fig. 5D, E). Independent networks for each lncRNA were constructed to highlight its linked DEGs in the PPs and pancreatic islets (Fig. 6). Multiple DEGs were commonly correlated to a specific lncRNA in both stages (Fig. 6). Furthermore, we identified 8 lncRNAs that were upregulated in PPs and pancreatic islets, including *AC108865.2*, *PANCR*, *AP001528.1*, *LINC02732*, *LINC01357*, *AC111000.4*, *AP000547.3*, and *AC140479.5* (Table 3). The correlation analysis revealed that only *AC111000.4* had Pearson correlation coefficients > 0.3, especially with *ABCC2* TF.

**Table 3:** Commonly dysregulated lncRNAs in the PPs and iPSC derived pancreatic islets from *FOXA2*^*−/−*^ iPSCs in comparison to that derived from WT-iPSCs

	**Pancreatic progenitors (PPs)**		**β-cell stage**	
**LncRNA**	**Log2 FC**	***p*-value**	**Log2 FC**	***p*-value**
**Downregulated**				
LINC02864	-4.972279036	0.000123222	-2.98476449	0.002945274
MEG3	-4.373714533	0.004838358	-1.695537434	8.06821E-05
AL133325.3	-3.99598566	0.0026067	-2.737498389	0.000396846
H19	-3.36547207	0.00485596	-1.050888105	0.006823965
ZNF667-AS1	-3.243039847	6.19752E-05	-2.717560856	0.000210262
CU634019.6	-3.194862483	0.039922842	-1.192479958	0.008498683
AC069277.1	-3.114859224	9.29544E-05	-2.679374923	0.000701003
LINC00543	-3.09914005	0.001683363	-1.29696446	0.001586351
LINC00261	-3.075824167	0.016451323	-1.650881593	9.75487E-05
AL161908.1	-2.757967046	0.002944123	-1.200190615	0.026481698
AC097639.1	-2.647646886	0.002050943	-1.541881067	0.01047655
AL035661.1	-2.278195817	0.004812208	-2.108152808	0.002375826
AC092171.3	-2.272636845	0.000741109	-1.147031159	0.000704907
MIR31HG	-2.222944778	0.02063138	-1.301936215	0.027976252
FAM215B	-2.169039549	0.015553135	-1.089686542	0.017146332
AC011503.2	-2.152742951	0.000603108	-1.970352528	9.47392E-05
AC073046.1	-2.132161904	0.006396904	-1.286680951	0.017689242
SLC25A25-AS1	-1.9302323	0.025532882	-1.05349219	0.008940334
AC048382.2	-1.858513511	0.001210312	-1.164296471	0.030130827
AC244636.3	-1.804998996	0.011401985	-1.102553403	0.000384212
AC016590.3	-1.64726758	0.01512032	-1.656663538	0.000219614
AC018521.6	-1.569659776	0.004432069	-1.147751692	0.011001064
AL591684.2	-1.46522189	0.006228583	-1.097860713	0.012037403
U73166.1	-1.429991468	0.029312295	-1.570767863	0.007890927
AL512625.1	-1.352837672	0.018258575	-1.04791555	0.002257768
AC006116.11	-1.27904533	0.005817342	-1.230767621	0.040102747
ZNF790-AS1	-1.229759258	0.007887571	-1.352811357	0.00145091
AL021395.1	-1.22320025	0.021478149	-1.456225426	0.000491092
MNX1-AS2	-1.220972989	0.016022764	-1.119116833	0.011214612
AC091563.1	-1.142586895	0.002025041	-1.852901184	0.000864753
AL121761.1	-1.096367847	0.013523216	-2.47631697	0.002825188
AC007403.1	-1.083637207	0.015727536	-1.197241732	0.000293157
AC244197.2	-1.061029505	0.0288463	-1.701001108	0.004129482
AC122129.1	-1.053981503	0.007871661	-1.338206292	0.01405125
LINC01806	-1.023333368	0.023463599	-1.207405254	0.013336001
**Upregulated**				
AC108865.2	2.76635816	0.02203366	2.16623553	0.01491233
PANCR	1.68884068	0.00676697	1.32679449	0.04815831
AP001528.1	1.63797665	0.00049588	1.31355731	0.00045822
LINC02732	1.52230916	0.03275826	1.50124372	0.02094184
LINC01357	1.40909594	0.01116811	1.08663066	0.00471372
AC111000.4	1.36919958	0.04373589	1.70320464	0.00042295
AP000547.3	1.28967432	0.00223951	1.14135645	0.00160776
AC140479.5	1.14460205	0.030656	1.10003456	0.00085757

### Validation of the dysregulated lncRNAs in iPSC-derived pancreatic progenitors and islets

**Fig. 7:**
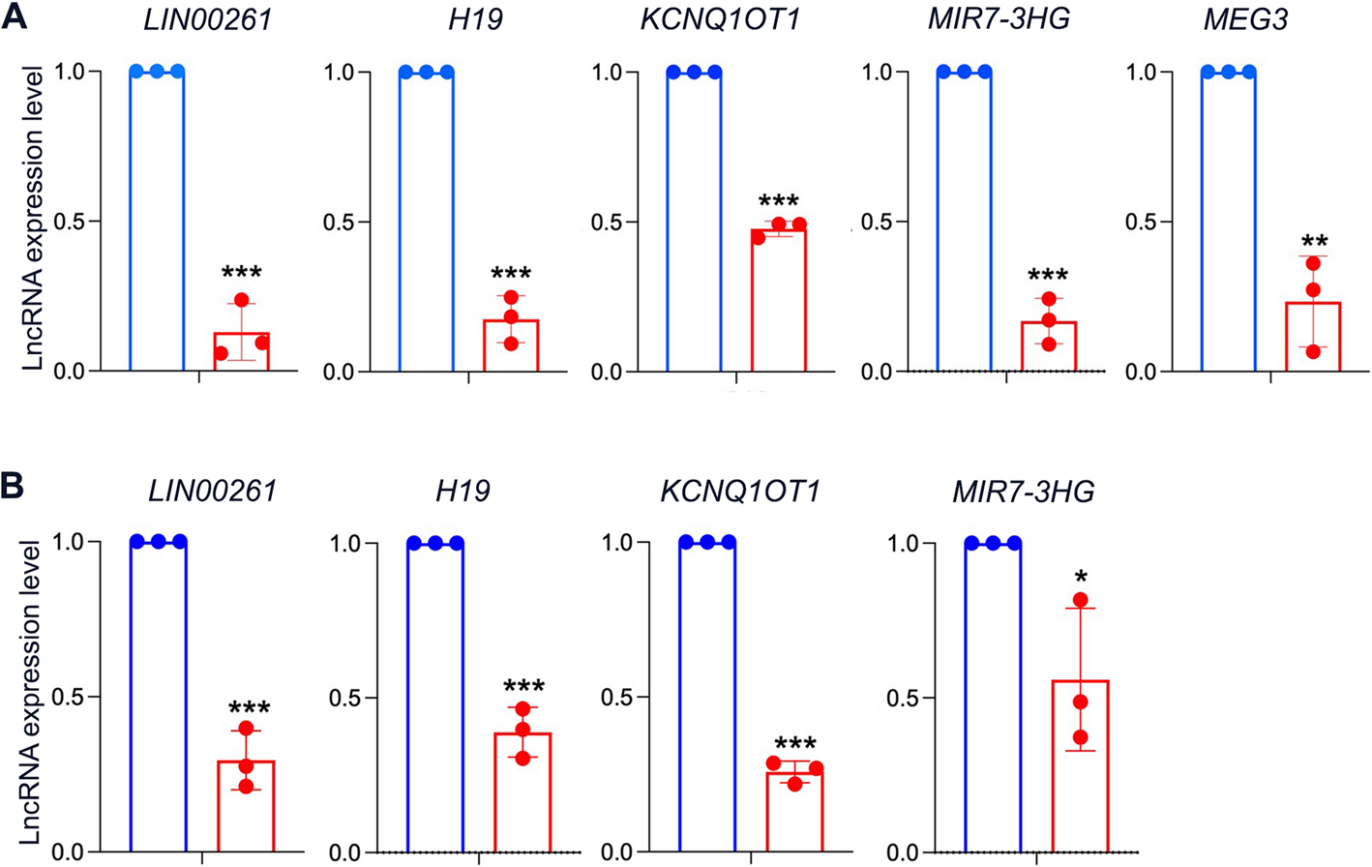
RT-qPCR validation for DE-lncRNAs crucial for pancreatic development and function. **A** RT-qPCR analysis for validation of selected DE-lncRNAs in iPSC-derived pancreatic progenitors (PPs), including *MEG3*, *H19*, *LINC00261*, *MIR7-3HG*, and *KCNQ1OT1* for pancreatic progenitors. **B** RT-qPCR analysis for validation of selected DE-lncRNAs in iPSC-derived pancreatic islet stage, including *H19*, *LINC00261, MIR7-3HG*, and *KCNQ1OT1.* Data are represented as mean ± SD; *p < 0.05, **p < 0.01, ***p < 0.001

To validate the DE-lncRNAs, we performed RT-qPCR on pancreatic progenitors and pancreatic islets. RT-qPCR analysis showed that the expression of lncRNA transcripts, including *MEG3*, *H19*, *LINC00261*, *MIR7-3HG*, and *KCNQ1OT1* was significantly downregulated in PPs derived from *FOXA2*^*−/−*^iPSCs compared to WT controls (Fig. 7A). Furthermore, at the islet stage, the expression of *H19*, *LINC00261, MIR7-3HG*, and *KCNQ1OT1* was significantly downregulated in pancreatic islets lacking FOXA2 compared to WT controls (Fig. 7B).

## Discussion

Differentiation of hPSCs into pancreatic islets is controlled by the expression of key genes and TFs that are specific for each stage during pancreatic development (as reviewed in [2]. Our recent study demonstrated that the absence of FOXA2 in iPSCs results in impaired differentiation into pancreatic islets, as evidenced by a notable decrease in the expression of pancreatic developmental genes [3]. Furthermore, we found that those downregulated genes are targets for several upregulated miRNAs in PPs lacking FOXA2 [39]. In this study, we employed the same iPSC model to examine the effect of FOXA2 depletion on the lncRNA profile at pancreatic progenitor and pancreatic islet stages. Our findings revealed that the alterations in the mRNA profiles linked to FOXA2 were accompanied with significant alterations in the expression of lncRNAs at both stages.

By analyzing RNA-seq results from PPs and pancreatic islets derived from WT-iPSCs and *FOXA2*^−/−^iPSCs, we observed a decrease in the expression of critical pancreatic genes involved in the development and function of pancreatic islets, such as *PDX1, NKX6.1, NEUROG3, NEUROD1, NKX2.2, INS, GCG,* and others [39]. We conducted a network analysis combining these downregulated pancreatic genes with DE-lncRNAs. This analysis revealed that 195 and 86 DE-lncRNAs in the PPs and pancreatic islets, respectively, were strongly correlated with genes known to regulate the development and function of pancreatic islets. Moreover, we identified 12 DE-lncRNAs that exhibited decreased expression in both PPs and pancreatic islets derived from *FOXA2*^*−/−*^iPSCs. These DE-lncRNAs include *MEG3*, *H19*, *ZNF667-AS1*, *LINC00543*, *LINC00261*, *AC097639.1*, *AL035661.1*, *SLC25A25-AS1*, *U73166.1*, *ZNF790-AS1*, *MNX1-AS2*, and *AC091563.1*. Some of those lncRNAs play important role in regulating the development and/or function of pancreatic islets. For example, *MEG3* plays a crucial role in controlling pancreatic β-cell mass and regulating the expression of Pdx1, Mafa, and Ins2, which are vital for pancreas development and insulin secretion [44, 45]. *MEG3* binding has been observed in the promotor region of FOXA2, and its depletion has been linked to reduced FOXA2 expression [46]. *MEG3* enhancer has been found to bind by FOXA2, PDX1, and NKX2.2 in human β-cells [47]. Moreover, the maternally expressed *H19* lncRNA has been shown to promote β-cell development by its close association with the *insulin-like growth factor 2 (Igf2*) locus [48, 49]. Intriguingly, our data confirmed this strong correlation, as *IGF2-AS* was significantly downregulated, particularly in the PPs stage. FOXA2 has been found to interact with the *H19* E1 enhancer, regulating its expression, particularly in the early developmental stage of fetal liver [50]. Additionally, we found that *LINC00261* was significantly downregulated and displayed a robust correlation with several pancreatic genes, such as FOXA2, PDX1, NKX6.1, TCF7L2, MNX1, GLIS3, CACNA1A, and PLCB4. It has been previously reported in several studies that *LINC00261* positively regulates FOXA2 [36, 37, 51], and the loss of *LINC00261* during pancreatic endocrine differentiation has been found to reduce the percentage of the generated insulin-secreting cells [38]. Moreover, we noticed a significant correlation between FOXA2 and SLC25A25-AS1 lncRNA. Downregulation of SLC25A25-AS1 has been linked to epithelial mesenchymal transition (EMT), resulting in the acquisition of mesenchymal characteristics [52], suggesting a possible epigenetic modulation of EMT-related lncRNAs by FOXA2. Another lncRNA that showed a strong correlation with FOXA2 and was downregulated in our study is SLC25A3, which its suppression has been reported to contribute to diabetes development by reducing ATP levels [53]. Furthermore, we identified MNX1-AS2, *ZNF667-AS1 (MORT*) and *ZNF790-AS1,* which were strongly correlated with FOXA2. MNX1 and ZNF are essential for pancreatic development [2, 54]. We also found that several downregulated lncRNAs, such as *LINC00543*, *AC097639.1*, *AL035661.1*, *U73166.1*, and *AC091563.1*, have not been investigated in the context of pancreatic development and their correlation with FOXA2 should be explored in future studies. Taken together, these findings suggest that lncRNAs may play a role in regulating the expression of key genes involved in pancreatic development, including FOXA2 and vice versa.

Several studies have reported that changes in the expression of lncRNAs are linked to pancreatic islet development and various types of diabetes. lncRNAs have been identified to play a role in the development of diabetes and may be potential biomarkers for the early detection of diabetes [55–57]. *LINC00261* and *MIR7-3HG* have been shown to be highly expressed lncRNAs in hESC-derived PPs in recent profiling studies [38]. Our study further supports the correlation between FOXA2 and these lncRNAs by demonstrating that their expression levels were decreased upon FOXA2 deletion, indicating their significance in pancreatic progenitor and β-cell development. Moreover, our findings demonstrated that several lncRNAs, downregulated upon FOXA2 deletion are known to be linked to diabetes. For instance, *MEG3* expression is strongly associated with diabetes, and its expression is downregulated in pancreatic islets of type 1 (T1D) and type 2 diabetes (T2D) in mouse and human [44, 58, 59]. Decreased expression of lncRNA *H19* has also been noticed in diabetes and linked to its clinicopathological abnormalities [60, 61]. Downregulation of *KCNQ1OT1* is associated with an increased risk of T2D [62, 63]. *GAS5* lncRNA is decreased in the serum of patients with diabetes and individuals with low *GAS5* levels are more susceptible to develop diabetes [64], as its reduction decreases the insulin secretion and increases the β-cell dysfunctionality [65]. Downregulation of lncRNA *TUG1* expression alters insulin secretion and induces apoptosis in pancreatic β-cells [66]. *MALAT1* downregulates the PDX1 expression and impairs the β-cell function [23], and the upregulation of *MALAT1* has been reported in the blood of T2D patients [67, 68]. *Linc13* is associated with T1D through its contribution in the inflammatory pathway activation and β-cell destruction [69]. Increased expression of lncRNA *PVT1* activates the oxidative stress and β-cell apoptosis; however, its silencing enhances the insulin secretory capacity [70], which classify the *PVT1* as a diabetes-associated lncRNA. A previous report has indicated that a heterozygous missense variant in FOXA2 can cause monogenic diabetes [8]. Also, the risk alleles for T2D have been found to be associated with the FOXA2 binding enhancer [7]. When take in conjunction with our recent findings demonstrating that deficiency of FOXA2 impairs pancreatic islet development and may lead to diabetes, these observations suggest the involvement of the alterations in lncRNA expression reported in this study may play a role in this effect.

To summarize, this study demonstrates that the deletion of FOXA2 disrupts the profiles of mRNAs and lncRNAs during pancreatic progenitor and β-cell stages of pancreatic development. Furthermore, this study revealed a strong association between lncRNAs and several critical pancreatic genes and TFs during pancreatic differentiation. The presented data suggest that the observed impairment in pancreatic islet development in the absence of FOXA2 is linked to significant changes in the expression profile of lncRNAs. Together with prior research, these findings suggest that dysregulated lncRNAs may play an essential role in the development and function of pancreatic islet cells and may contribute to the development of diabetes. Whether FOXA2 directly regulates the expression of the identified lncRNAs, or loss of FOXA2 and subsequent impairment of pancreatic development leads to deregulated lncRNA expression remains to be investigated. These findings provide a basis for further functional investigation into the newly identified lncRNAs to gain a better understanding of their role during pancreatic development.

### Supplementary Information

**Additional file 1: Supplementary Fig. 1.** Differentiation of FOXA2 knockout iPSCs into pancreatic islets. (A) A diagram showing the differentiation protocol used in this study. FOXA2-/- iPSCs and WT- 2 iPSCs were differentiated into pancreatic progenitors (PPs), endocrine progenitors (EPs), and pancreatic islet cells. The initial four stages of differentiation were generated using our protocol, while the subsequent stages of islet differentiation followed Rezania protocol. (B) In comparison to the WT control, the differentiation of FOXA2-/- iPSCs demonstrated a significant reduction in the expression of PP markers, including PDX1 and NKX6.1. (C) The Absence of FOXA2 resulted in a notable decrease in the expression of endocrine progenitor markers, NGN3 and NKX2.2. (D) Loss of FOXA2 led to a substantial reduction in the expression of insulin (INS) and glucagon (GCG), indicating a decline in both beta and alpha cell mass within the derived pancreatic islets. **Supplementary Fig. 2.** Heatmaps for the correlated downregulated lncRNAs in pancreatic progenitors. Separate heatmaps are displayed for the correlation of lncRNAs with FOXA2, PDX1, and NKX6.1, as well as a heatmap for lncRNAs that are commonly correlated with FOXA2, PDX1, and NKX6.1. **Supplementary Fig. 3.** Co-expression network analysis of downregulated lncRNAs and DEGs in pancreatic progenitors derived from FOXA2-/- iPSCs. A network showing the correlation analysis between the downregulated DE-lncRNAs and DEGs in the iPSC-derived pancreatic progenitors (PPs) lacking FOXA2. **Supplementary Fig. 4.** Strongly upregulated lncRNAs with a Pearson correlation coefficient (PC) > 0.3 in the pancreatic progenitors derived from iPSCs lacking FOXA2. The upregulated lncRNAs are presented in a heatmap (A), and their correlation with DEGs is depicted in a network using expression data from an online database of 305 pancreatic tissues (B) and data generated in this study from iPSC-derived pancreatic islets (C). **Supplementary Fig. 5.** Co-expression network analysis of downregulated lncRNAs and DE-mRNAsin pancreatic islets derived from FOXA2-/- iPSCs. The correlation analysis between the downregulated DE-lncRNAs and DE-mRNAs in iPSC-derived pancreatic islets lacking FOXA2. **Supplementary Fig. 6.** Heatmaps for the downregulated lncRNAs that are correlated in the pancreatic islets. Separate heatmaps are displayed for the correlation of lncRNAs with FOXA2, PDX1, NKX6.1, and ABCC8, as well as a heatmap for lncRNAs that are commonly correlated with FOXA2, PDX1, NKX6.1 and ABCC8. **Supplementary Fig. 7.** Strongly upregulated lncRNAs with a Pearson correlation coefficient (PC) > 0.3 in the pancreatic islets derived from iPSCs lacking FOXA2. The upregulated lncRNAs are presented in a heatmap (A), and their correlation with DEGs is depicted in a network (B). **Supplementary Fig. 8.** Co-expression network analysis of upregulated lncRNAs and DEmRNAs in pancreatic islets derived from FOXA2-/- iPSCs. The correlation analysis between the upregulated DE-lncRNAs and DE-mRNAs in iPSC-derived pancreatic islets lacking FOXA2.

**Additional file 2: Supplementary Table 1.** List of primers used for qPCR validation of selected deferentially expressed lncRNAs in the pancreatic progenitors and islets derived from FOXA2-/- iPSCs in comparison to those derived from WT-iPSCs. **Supplementary Table 2.** Top dysregulated lncRNAs in pancreatic progenitors (PPs) derived from FOXA2-/- iPSCs in comparison to those derived from WT-iPSCs (*p* < 0.05). **Supplementary Table 3.** Selected differentially expressed genes (DEGs) to study its correlation to the lncRNAs. **Supplementary Table 4.** List of downregulated lncRNAs with common strong correlation to FOXA2, PDX1 and NKX6.1 with a Pearson correlation (PC) > 0.3 in the pancreatic progenitors (PPs) derived from FOXA2-/- iPSCs in comparison to those derived from WT-iPSCs (*p* < 0.05). **Supplementary Table 5.** Top dysregulated lncRNAs in pancreatic islets derived from FOXA2-/- iPSCs in comparison to those derived from WT-iPSCs (*p* < 0.05). **Supplementary Table 6.** List of downregulated lncRNAs with common strong correlation to FOXA2, PDX1, NKX6.1, and ABCC8 with a Pearson correlation (PC) > 0.3 in the pancreatic islets derived from FOXA2 -/- iPSCs in comparison to those derived from WT-iPSCs (*p* < 0.05).

## Data Availability

The data that support the findings of this study are available from the corresponding author upon reasonable request.
