# Supplementary material for: Genome-wide differential expression profiling of long non-coding RNAs in FOXA2 knockout iPSC-derived pancreatic cells
Source: Cell Commun Signal. 2023 Sep 5;21:229. doi: 10.1186/s12964-023-01212-2 (PMC10478503; doi:10.1186/s12964-023-01212-2)
Supplement: Supplementary file 2 — Additional file 1: Supplementary Fig. 1. Differentiation of FOXA2 knockout iPSCs into pancreatic islets. (A) A diagram showing the differentiation protocol used in this study. FOXA2-/- iPSCs and WT- 2 iPSCs were differentiated into pancreatic progenitors (PPs), endocrine progenitors (EPs), and pancreatic islet cells. The initial four stages of differentiation were generated using our protocol, while the subsequent stages of islet differentiation followed Rezania protocol. (B) In comparison to the WT control, the differentiation of FOXA2-/- iPSCs demonstrated a significant reduction in the expression of PP markers, including PDX1 and NKX6.1. (C) The Absence of FOXA2 resulted in a notable decrease in the expression of endocrine progenitor markers, NGN3 and NKX2.2. (D) Loss of FOXA2 led to a substantial reduction in the expression of insulin (INS) and glucagon (GCG), indicating a decline in both beta and alpha cell mass within the derived pancreatic islets. Supplementary Fig. 2. Heatmaps for the correlated downregulated lncRNAs in pancreatic progenitors. Separate heatmaps are displayed for the correlation of lncRNAs with FOXA2, PDX1, and NKX6.1, as well as a heatmap for lncRNAs that are commonly correlated with FOXA2, PDX1, and NKX6.1. Supplementary Fig. 3. Co-expression network analysis of downregulated lncRNAs and DEGs in pancreatic progenitors derived from FOXA2-/- iPSCs. A network showing the correlation analysis between the downregulated DE-lncRNAs and DEGs in the iPSC-derived pancreatic progenitors (PPs) lacking FOXA2. Supplementary Fig. 4. Strongly upregulated lncRNAs with a Pearson correlation coefficient (PC) > 0.3 in the pancreatic progenitors derived from iPSCs lacking FOXA2. The upregulated lncRNAs are presented in a heatmap (A), and their correlation with DEGs is depicted in a network using expression data from an online database of 305 pancreatic tissues (B) and data generated in this study from iPSC-derived pancreatic islets (C). Supplementary Fig. 5. Co [file 12964_2023_1212_MOESM1_ESM.pdf]

Supplementary Figures

Supplementary Figure 1

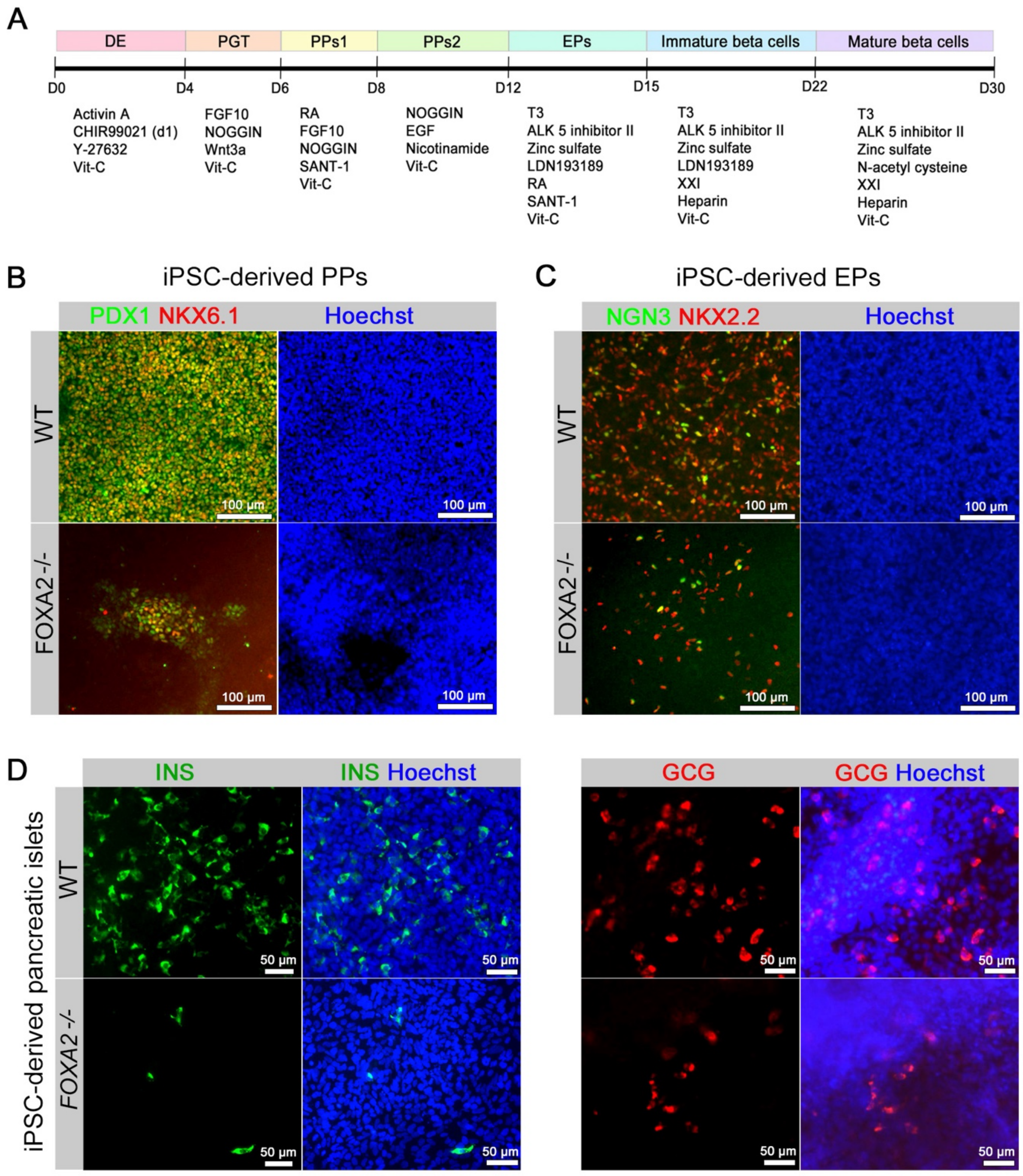

Supplementary Figure 1. Differentiation of FOXA2 knockout iPSCs into pancreatic islets. (A) A diagram showing the differentiation protocol used in this study. *FOXA2*$^{-/-}$iPSCs and WT-

iPSCs were differentiated into pancreatic progenitors (PPs), endocrine progenitors (EPs), and pancreatic islet cells. The initial four stages of differentiation were generated using our protocol, while the subsequent stages of islet differentiation followed Rezania protocol. (B) In comparison to the WT control, the differentiation of *FOXA2*$^{-/-}$iPSCs demonstrated a significant reduction in the expression of PP markers, including PDX1 and NKX6.1. (C) The Absence of FOXA2 resulted in a notable decrease in the expression of endocrine progenitor markers, NGN3 and NKX2.2. (D) Loss of FOXA2 led to a substantial reduction in the expression of insulin (INS) and glucagon (GCG), indicating a decline in both beta and alpha cell mass within the derived pancreatic islets.

Supplementary Figure 2

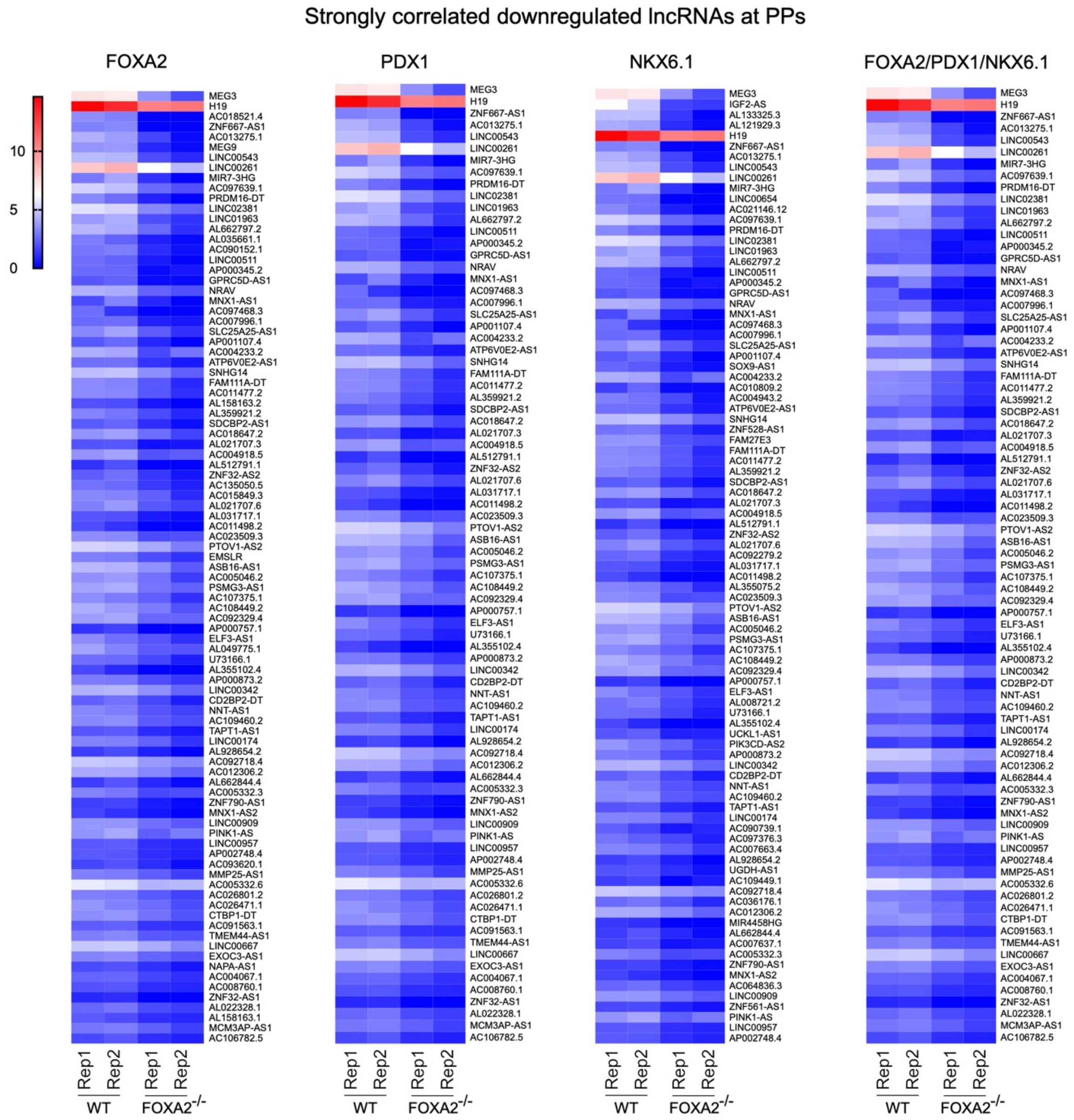

Supplementary Figure 2. Heatmaps for the correlated downregulated lncRNAs in pancreatic progenitors. Separate heatmaps are displayed for the correlation of lncRNAs with FOXA2, PDX1, and NKX6.1, as well as a heatmap for lncRNAs that are commonly correlated with FOXA2, PDX1, and NKX6.1.

Supplementary Figure 3

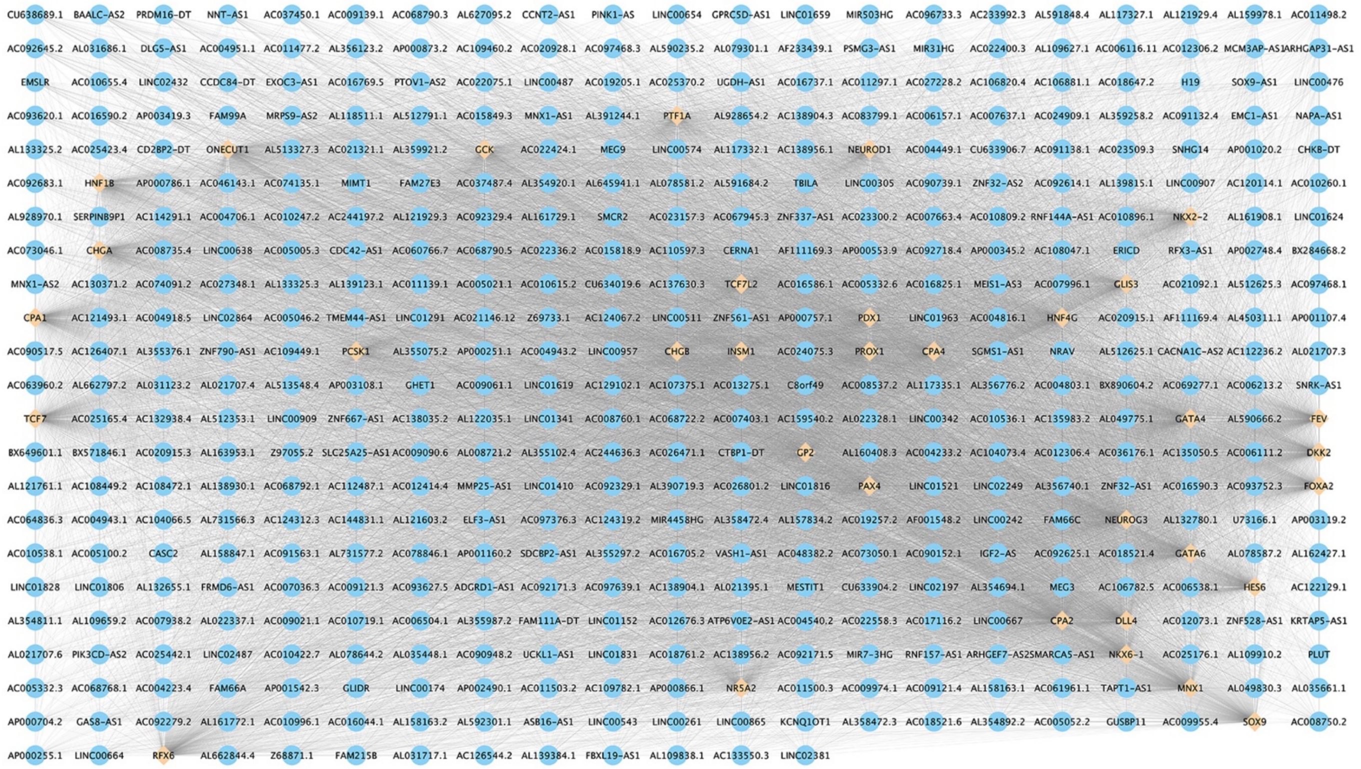

Supplementary Figure 3. Co-expression network analysis of downregulated lncRNAs and DEGs in pancreatic progenitors derived from *FOXA2*$^{-/-}$ iPSCs. A network showing the correlation analysis between the downregulated DE-lncRNAs and DEGs in the iPSC-derived pancreatic progenitors (PPs) lacking FOXA2.

Supplementary Figure 4

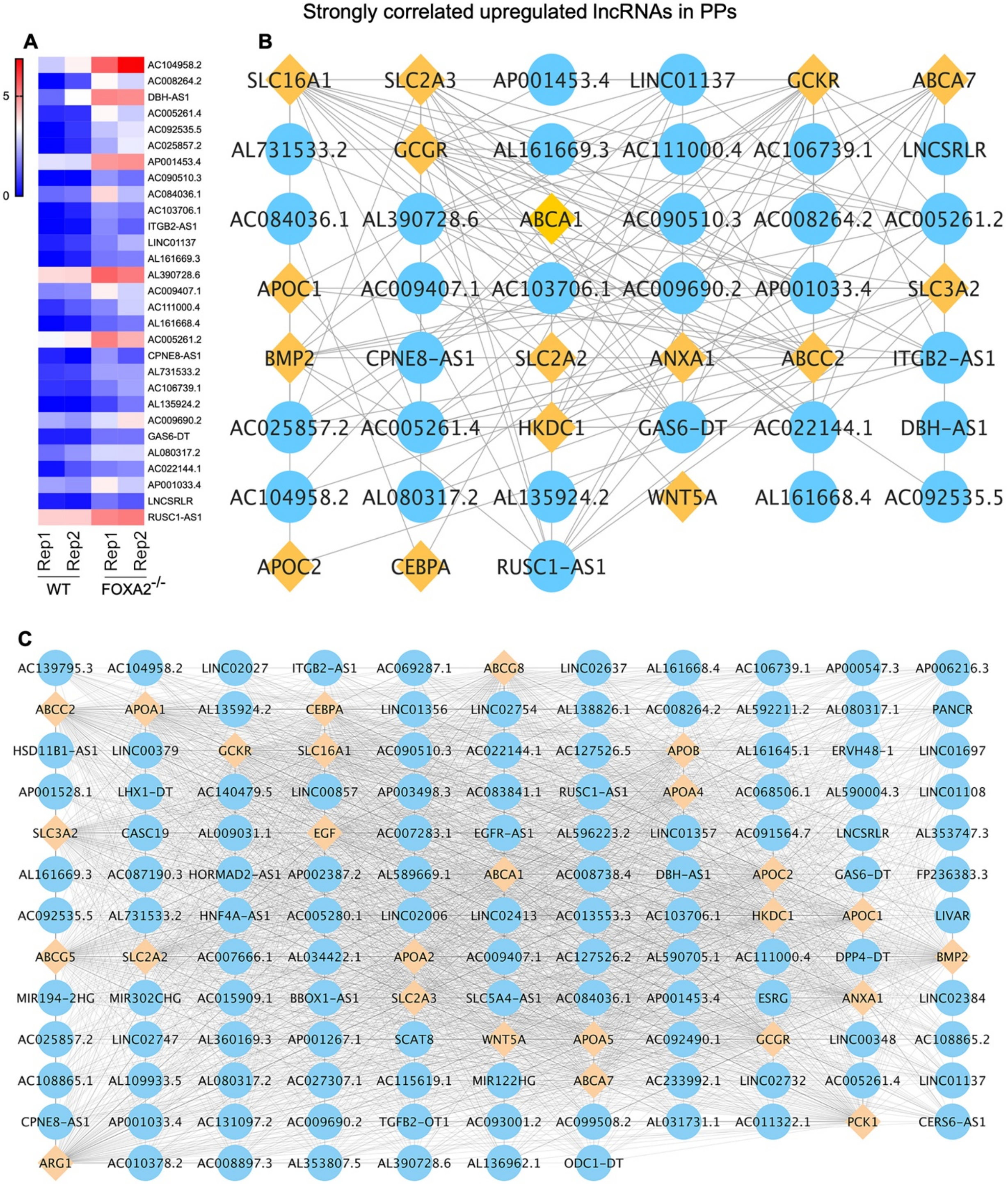

Supplementary Figure 4. Strongly upregulated lncRNAs with a Pearson correlation coefficient (PC) >0.3 in the pancreatic progenitors derived from iPSCs lacking FOXA2. The upregulated lncRNAs are presented in a heatmap (A), and their correlation with DEGs is depicted in a network using expression data from an online database of 305 pancreatic tissues (B) and data generated in this study from iPSC-derived pancreatic islets (C).

Supplementary Figure 5

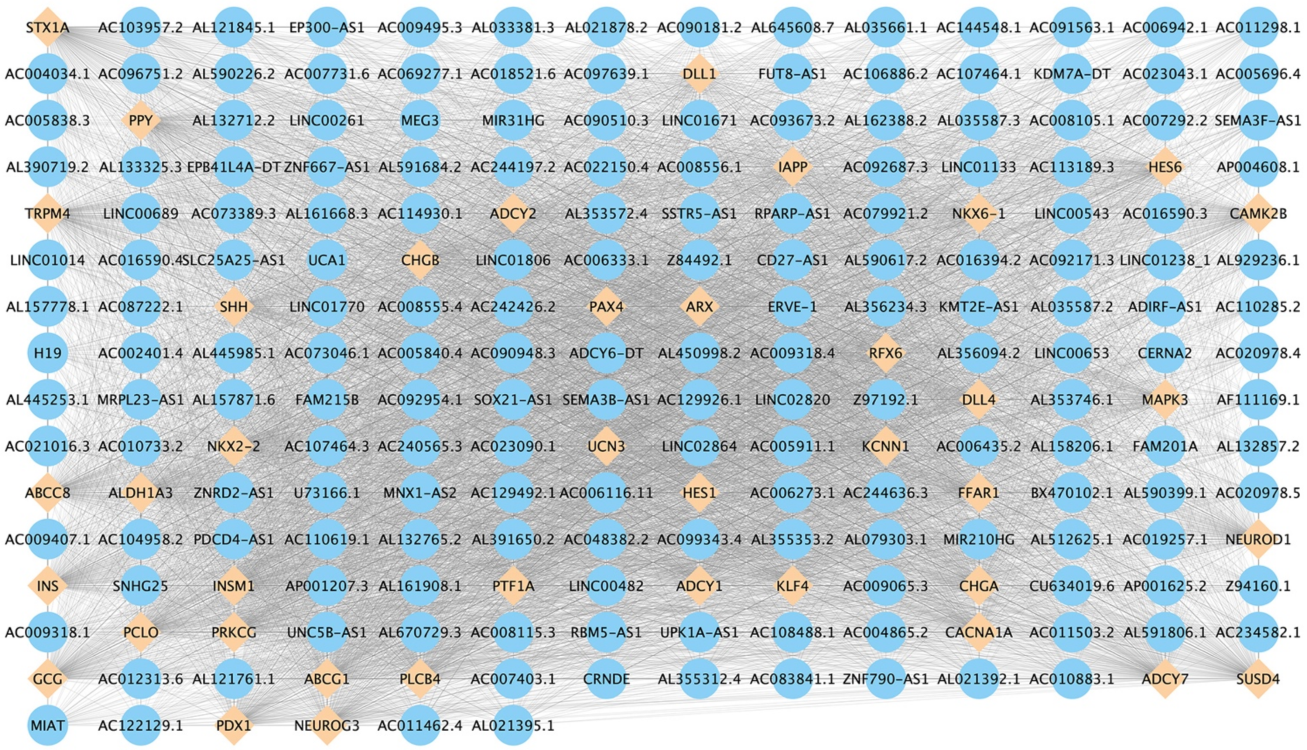

Supplementary Figure 5. Co-expression network analysis of downregulated lncRNAs and DE-mRNAs in pancreatic islets derived from *FOXA2*$^{-/-}$ iPSCs. The correlation analysis between the downregulated DE-lncRNAs and DE-mRNAs in iPSC-derived pancreatic islets lacking FOXA2.

Supplementary Figure 6

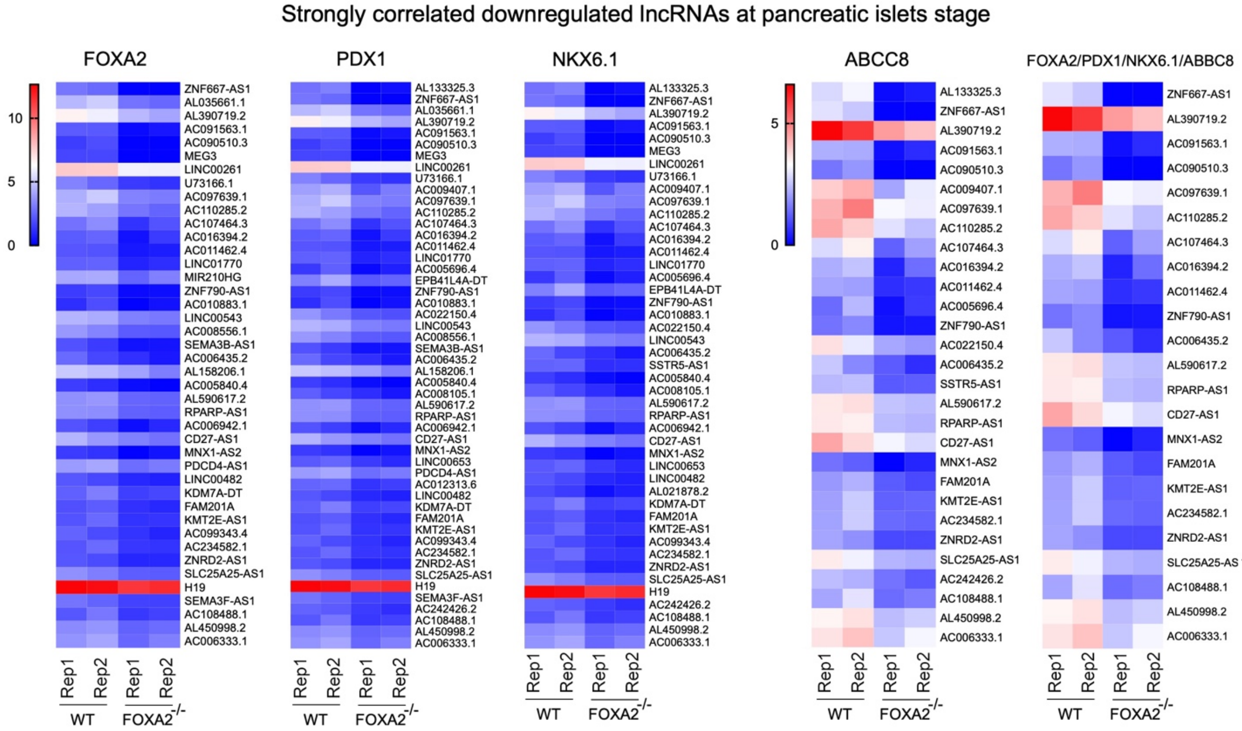

Supplementary Figure 6. Heatmaps for the downregulated lncRNAs that are correlated in the pancreatic islets. Separate heatmaps are displayed for the correlation of lncRNAs with FOXA2, PDX1, NKX6.1, and ABCC8, as well as a heatmap for lncRNAs that are commonly correlated with FOXA2, PDX1, NKX6.1 and ABCC8.

Supplementary Figure 7

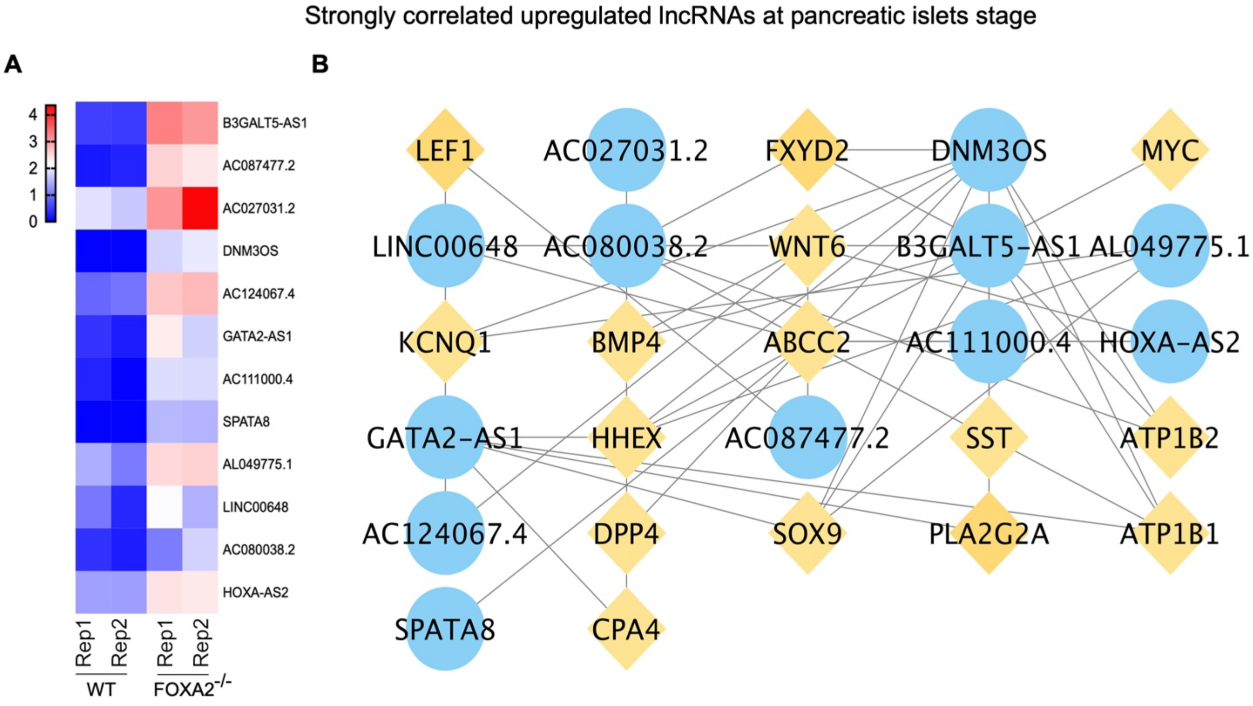

Supplementary Figure 7. Strongly upregulated lncRNAs with a Pearson correlation coefficient (PC) >0.3 in the pancreatic islets derived from iPSCs lacking FOXA2. The upregulated lncRNAs are presented in a heatmap (A), and their correlation with DEGs is depicted in a network (B).

Supplementary Figure 8

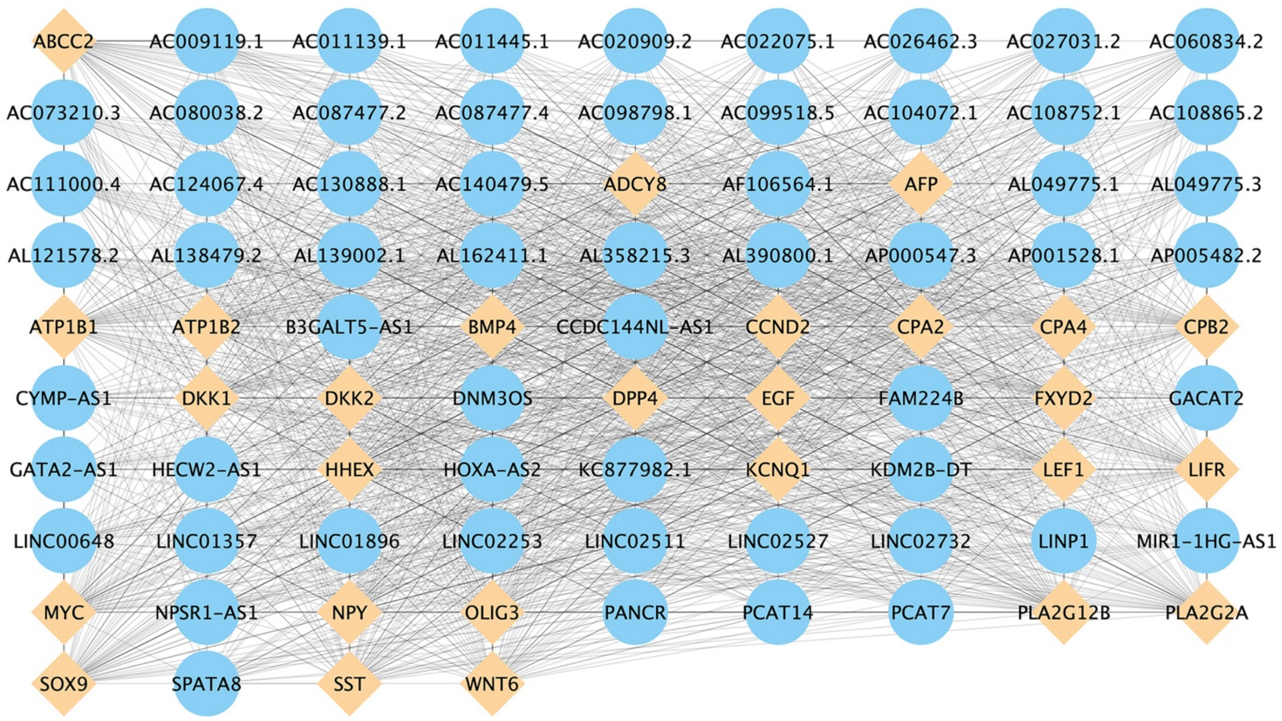

Supplementary Figure 8. Co-expression network analysis of upregulated lncRNAs and DE-mRNAs in pancreatic islets derived from *FOXA2*$^{-/-}$ iPSCs. The correlation analysis between the upregulated DE-lncRNAs and DE-mRNAs in iPSC-derived pancreatic islets lacking FOXA2.
