# Supplementary material for: Genome-wide differential expression profiling of long non-coding RNAs in FOXA2 knockout iPSC-derived pancreatic cells
Source: Cell Commun Signal. 2023 Sep 5;21:229. doi: 10.1186/s12964-023-01212-2 (PMC10478503; doi:10.1186/s12964-023-01212-2)
Supplement: Supplementary file 3 — Additional file 2: Supplementary Table 1. List of primers used for qPCR validation of selected deferentially expressed lncRNAs in the pancreatic progenitors and islets derived from FOXA2-/- iPSCs in comparison to those derived from WT-iPSCs. Supplementary Table 2. Top dysregulated lncRNAs in pancreatic progenitors (PPs) derived from FOXA2-/- iPSCs in comparison to those derived from WT-iPSCs (p < 0.05). Supplementary Table 3. Selected differentially expressed genes (DEGs) to study its correlation to the lncRNAs. Supplementary Table 4. List of downregulated lncRNAs with common strong correlation to FOXA2, PDX1 and NKX6.1 with a Pearson correlation (PC) > 0.3 in the pancreatic progenitors (PPs) derived from FOXA2-/- iPSCs in comparison to those derived from WT-iPSCs (p < 0.05). Supplementary Table 5. Top dysregulated lncRNAs in pancreatic islets derived from FOXA2-/- iPSCs in comparison to those derived from WT-iPSCs (p < 0.05). Supplementary Table 6. List of downregulated lncRNAs with common strong correlation to FOXA2, PDX1, NKX6.1, and ABCC8 with a Pearson correlation (PC) > 0.3 in the pancreatic islets derived from FOXA2 -/- iPSCs in comparison to those derived from WT-iPSCs (p < 0.05). [file 12964_2023_1212_MOESM2_ESM.pdf]

Supplementary Table 1. List of primers used for qPCR validation of selected deferentially expressed lncRNAs in the pancreatic progenitors and islets derived from *FOXA2*$^{-/-}$iPSCs in comparison to those derived from WT-iPSCs.

LncRNA	Forward	Reverse
H19	TCAGCTCTGGGATGATGTGGT	CTCAGGAATCGGCTCTGGAAG
KCNQ1OT1	TTCTGGAGGCGATTGAGGC	AGCAACCAGAACCAGGTGAGAG
MEG3	CTCCCCTTCTAGCGCTCACG	CTAGCCGCCGTCTATACTACCGGCT
LINC00261	TTCACAGGAAATGGCATCAA	GTGGCCTCAGAACCATCTTC
MIR7-3HG	TGGACATTTTCCCAGAAAGG	CAGGGTTCTGGAGACGAAAC

Supplementary Table 2. Top dysregulated lncRNAs in pancreatic progenitors (PPs) derived from *FOXA2*$^{-/-}$iPSCs in comparison to those derived from WT-iPSCs (p<0.05)

LncRNA Name	Log2 FC	p-value
**Downregulated**		
LINC02864	-4.972279036	0.000123222
MEG3	-4.373714533	0.004838358
AL138930.1	-4.15932484	0.001799866
AC126407.1	-4.155093726	0.001541997
IGF2-AS	-4.148365238	0.002702133
AL133325.3	-3.99598566	0.0026067
AC025423.4	-3.964876545	0.000183284
LINC00305	-3.859552674	0.004238662
AL121929.3	-3.416597619	0.001174283
AC061961.1	-3.388249594	0.003986678
H19	-3.36547207	0.00485596
AC018521.4	-3.32837027	0.000103837
AL162427.1	-3.319098026	0.000876486
ZNF667-AS1	-3.243039847	6.20E-05
AC013275.1	-3.199973442	0.008418639
CU634019.6	-3.194862483	0.039922842
MEG9	-3.127263317	0.00098301
AC069277.1	-3.114859224	9.30E-05

LINC00543	-3.09914005	0.001683363
LINC00261	-3.075824167	0.016451323
AC108472.1	-3.062423014	0.000403933
MIR7-3HG	-2.893704218	0.014703189
AC007938.2	-2.891740646	0.003007248
LINC01831	-2.81775842	0.005476339
LINC00654	-2.809462826	6.40E-05
AC092171.5	-2.780893479	0.006553328
AL161908.1	-2.757967046	0.002944123
MIR503HG	-2.744943277	0.002853921
AC010247.2	-2.741236062	0.009904781
AC021146.12	-2.710706091	0.002958143
AC016769.5	-2.702911977	0.013500486
LINC02197	-2.691766808	0.001114645
AC097639.1	-2.647646886	0.002050943
PRDM16-DT	-2.579795911	0.005966927
CU638689.1	-2.578260122	0.033434325
AC144831.1	-2.558576269	0.032584046
AL133325.2	-2.519513031	0.006098968
AC124312.3	-2.513513653	0.001853611
AC022424.1	-2.500425873	0.023114378
LINC00242	-2.497374664	0.000616074
LINC02381	-2.495033889	0.00483467
FAM99A	-2.49501718	0.0355912
AC130371.2	-2.49353626	0.008468086
LINC01963	-2.455544319	0.003180806
AC074135.1	-2.433789841	0.01440959
BAALC-AS2	-2.423625955	0.001725947
AL662797.2	-2.409764533	0.01791013
AC092614.1	-2.38036566	0.00135715
AC010615.2	-2.361375188	0.007314077
AF111169.3	-2.335452742	0.018354238
AL450311.1	-2.327553203	0.036962968
AC097468.1	-2.290565215	0.000867715
AL035661.1	-2.278195817	0.004812208
AC090152.1	-2.276363099	0.003367408
AC092171.3	-2.272636845	0.000741109
AC114291.1	-2.26982974	0.0319982
LINC01152	-2.265165856	0.014965185
AC011297.1	-2.256575303	0.000370759
AC018761.2	-2.252761823	0.025166105
LINC00664	-2.247963398	0.001271512
AC027228.2	-2.242915535	0.006087661
LINC00511	-2.227202614	0.002100061
MIR31HG	-2.222944778	0.02063138
AP000345.2	-2.209008733	0.000570614
AL355376.1	-2.199696784	0.002193172
AC022075.1	-2.191635173	0.001516008
FAM215B	-2.169039549	0.015553135
AC011503.2	-2.152742951	0.000603108
AC092329.1	-2.152096255	0.004297716
CCDC84-DT	-2.133562032	0.000151545
AC073046.1	-2.132161904	0.006396904

LINC01828	-2.124075894	0.000170202
GPRC5D-AS1	-2.087789286	0.000429436
NRAV	-2.086094618	0.001259205
MNX1-AS1	-2.081998069	0.038034509
AL109838.1	-2.060172052	0.011385625
VASH1-AS1	-2.053151617	0.015418298
AC016705.2	-2.03479919	0.037247535
LINC01624	-2.032184587	0.002435727
TBILA	-2.011886891	0.012318416
AC073050.1	-2.006338516	0.00168343
**Upregulated**		
AL009031.1	4.724394553	0.000834619
AC115619.1	4.418593266	0.009993118
AP001267.1	3.98790887	0.000288377
HNF4A-AS1	3.678908482	0.011621532
LIVAR	3.378633167	0.000453156
AC108865.1	3.205817049	0.037893201
AC104958.2	3.066906112	0.010419581
AC068506.1	3.058578237	0.001514246
AC010378.2	3.051401033	0.000228374
LINC02413	2.834299599	0.012323499
HORMAD2-AS1	2.799693817	0.003526626
AC108865.2	2.766358162	0.022033663
LINC02027	2.694186894	0.039653842
ERVH48-1	2.660811947	0.003783403
AC008264.2	2.64584345	0.008036352
DBH-AS1	2.62489971	0.033448781
AC008897.3	2.593255581	0.007734204
LINC01108	2.546176988	0.015403468
AC005261.4	2.525968646	0.002745155
AC092535.5	2.463911972	0.003267989
LINC00379	2.458830361	0.003128015
AC092490.1	2.456948573	0.007553943
AC025857.2	2.412292869	0.006863213
SCAT8	2.396276506	0.043778305
TGFB2-OT1	2.329088357	0.049567226
MIR194-2HG	2.294492837	0.005166698
MIR122HG	2.282376717	0.001290674
CERS6-AS1	2.257234783	0.045748977
AL589669.1	2.149756813	0.000688266
AC027307.1	2.137114245	0.000189316
AC093001.2	2.119661145	0.010881781
AP003498.3	2.108538992	0.008970491
AC087190.3	2.103006433	0.042993789
AP006216.3	2.056113708	0.008471569

Supplementary Table 3. Selected differentially expressed genes (DEGs) to study its correlation to the lncRNAs

Selected downregulated DEGs in *FOXA2*$^{-/-}$PPs compared to those in WT-PPs		
**DEGs**	**Log2-FC**	**p-value**
FOXA2	-2.44875	0.009809
ONECUT1	-4.17368	0.00159
HNF1B	-2.28455	0.002219
HNF4G	-1.00013	0.008878
PDX1	-4.74807	0.036873
NKX6-1	-6.86535	0.000836
NKX2-2	-5.3127	0.000787
NEUROG3	-6.12046	0.000963
NEUROD1	-4.83833	0.004375
GATA4	-1.4989	0.010756
GATA6	-1.90134	0.005377
SOX9	-2.87154	0.004139
HES6	-3.3048	0.012777
MNX1	-2.57429	0.046898
PAX4	-5.5499	0.000672
RFX6	-3.66149	0.025842
TCF7L2	-1.17691	0.028179
TCF7	-1.09786	0.008595
PROX1	-2.31588	0.035846
GLIS3	-2.46738	0.006005
PTF1A	-4.67047	0.002167
CPA1	-5.87937	0.000994
CPA2	-6.8263	0.00137
CPA4	-1.39858	0.04519
CHGA	-7.69738	0.00276
CHGB	-2.21194	0.008774
MNX1	-2.57429	0.046898
FEV	-5.44357	0.000341
PCSK1	-2.12607	0.013956
DKK2	-5.01933	0.000881
DLL4	-2.25185	0.006545
INSM1	-5.65338	0.013488
GP2	-5.35935	0.001233
GCK	-2.44924	0.001482
NR5A2	-1.62177	0.00322

Selected upregulated DEGs in *FOXA2*$^{-/-}$PPs compared to those of WT-PPs		
**DEGs**	**Log2 FC**	**p-value**
ABCA1	1.605089	0.008066
ABCA7	1.158401	0.018869
ABCC2	3.465378	0.012955
ABCG5	3.23774	0.001113
ABCG8	1.930718	0.025729
ANXA1	2.432721	0.001357
APOA1	4.903439	0.042478
APOA2	5.853378	0.044592
APOA4	7.86569	0.029359
APOA5	2.558482	0.039732
APOB	5.423966	0.033082
APOC1	2.901518	0.048535
APOC2	7.225852	0.019385
ARG1	4.444276	0.007052
BMP2	1.423967	0.016743
CEBPA	2.671298	0.027329
EGF	2.198566	0.009222
GCGR	4.579549	0.004129
GCKR	2.619156	0.010879
HKDC1	3.909498	0.034437
PCK1	5.381311	0.000487
SLC16A1	1.20825	0.007184
SLC2A2	3.325715	0.009322
SLC2A3	3.615408	0.000233
SLC3A2	1.155469	0.035476
WNT5A	1.682946	0.002881

Selected downregulated DEGs in *FOXA2*$^{-/-}$pancreatic islet compared with WT		
**DEGs**	**Log2 FC**	**p-value**
ABCC8	-1.34188	0.004895
ABCG1	-1.79741	0.00102
ADCY1	-1.44581	0.002243
ADCY2	-1.3056	0.007542
ADCY7	-1.45526	0.035309
ALDH1A3	-2.44977	0.000733
ARX	-1.61254	0.045159
CACNA1A	-1.26132	0.004077
CAMK2B	-2.44143	0.000227
CHGA	-1.36455	0.026737
CHGB	-1.27697	0.024924
DLL1	-2.90846	0.000551
DLL4	-1.89145	0.001372
FFAR1	-1.3157	0.000939
GCG	-4.08287	0.000356
HES1	-1.28388	0.037253
HES6	-2.22305	0.02261
IAPP	-1.01456	0.018731
INS	-4.81394	0.000564
INSM1	-3.91923	0.00502
KCNN1	-1.89248	0.006227
KLF4	-1.82608	0.00713
MAPK3	-1.04656	0.002777
NEUROD1	-1.78887	0.006574
NEUROG3	-3.21965	0.003294
NKX2-2	-2.8967	0.008452
NKX6-1	-2.0084	0.020767
PAX4	-1.45515	0.046518
PCLO	-1.11105	0.032656
PDX1	-1.39198	0.004558
PLCB4	-1.00193	0.004344
PPY	-2.12992	0.006051
PRKCG	-1.74019	0.013747
PTF1A	-2.49643	0.022354
RFX6	-2.04219	0.001358
SHH	-2.13832	0.000222
STX1A	-1.13328	0.005714
SUSD4	-2.45104	0.001329
TRPM4	-1.89471	0.000285

Selected upregulated DEGs in *FOXA2*$^{-/-}$ pancreatic islets compared with WT ($p<0.05$)		
**DEGs**	**Log2 FC**	**p-value**

ATP1B1	1.010252	0.00333
ATP1B2	1.09996	0.021689
FXYD2	1.716178	0.008968
ADCY8	1.646068	0.023116
CPA2	2.492019	0.003022
CPB2	1.761378	0.010662
CPA4	1.743376	0.008392
PLA2G2A	2.781383	0.012291
PLA2G12B	1.577241	0.020816
KCNQ1	1.791418	0.00981
AFP	4.526164	0.000698
NPY	3.603045	0.001557
SST	1.532785	0.013788
HHEX	1.040459	0.02109
ABCC2	1.022091	0.021114
SOX9	1.474219	0.025428
EGF	1.858628	0.002687
BMP4	3.557951	0.000201
DPP4	2.635987	0.023592
OLIG3	3.438869	0.00091
WNT6	3.256271	0.010484
DKK1	1.516249	0.002554
DKK2	2.140417	0.009316
LIFR	1.044593	0.032018
LEF1	1.629145	0.01226
MYC	1.142383	0.036299
CCND2	1.51378	0.029444

Supplementary Table 4. List of downregulated lncRNAs with common strong correlation to FOXA2, PDX1 and NKX6.1 with a Pearson correlation (PC) > 0.3 in the pancreatic progenitors (PPs) derived from *FOXA2*$^{-/-}$iPSCs in comparison to those derived from WT-iPSCs (p<0.05).

LncRNA	Log2-FC	P-value	PC to FOXA2	PC to PDX1	PC to NKX6.1
MEG3	-4.3737145	0.00483836	0.518**	0.368**	0.376**
H19	-3.3654721	0.00485596	0.459**	0.404**	0.340**
ZNF667-AS1	-3.2430398	6.1975E-05	0.436**	0.587**	0.549**
AC013275.1	-3.1999734	0.00841864	0.504**	0.756**	0.637**
LINC00543	-3.0991401	0.00168336	0.636**	0.657**	0.516**
LINC00261	-3.0758242	0.01645132	0.347**	0.314**	0.361**
MIR7-3HG	-2.8937042	0.01470319	0.422**	0.547**	0.565**
AC097639.1	-2.6476469	0.00205094	0.377**	0.434**	0.356**
PRDM16-DT	-2.5797959	0.00596693	0.470**	0.583**	0.538**
LINC02381	-2.4950339	0.00483467	0.481**	0.538**	0.542**
LINC01963	-2.4555443	0.00318081	0.318**	0.499**	0.392**
AL662797.2	-2.4097645	0.01791013	0.402**	0.423**	0.388**
LINC00511	-2.2272026	0.00210006	0.365**	0.398**	0.380**
AP000345.2	-2.2090087	0.00057061	0.400**	0.350**	0.336**
GPRC5D-AS1	-2.0877893	0.00042944	0.394**	0.565**	0.505**
NRAV	-2.0860946	0.0012592	0.518**	0.671**	0.634**
MNX1-AS1	-2.0819981	0.03803451	0.421**	0.504**	0.457**
AC097468.3	-1.9842205	0.02932675	0.441**	0.423**	0.391**
AC007996.1	-1.9808234	0.00046053	0.492**	0.486**	0.457**
SLC25A25-AS1	-1.9302323	0.02553288	0.484**	0.453**	0.441**
AP001107.4	-1.928512	0.01123832	0.344**	0.493**	0.509**
AC004233.2	-1.8629106	0.01745867	0.410**	0.494**	0.500**
ATP6V0E2-AS1	-1.7973721	0.00439557	0.370**	0.419**	0.435**
SNHG14	-1.783735	0.02106934	0.393**	0.561**	0.513**
FAM111A-DT	-1.7449084	0.02667099	0.356**	0.460**	0.473**
AC011477.2	-1.714697	0.01645531	0.322**	0.402**	0.352**
AL359921.2	-1.675371	0.01584837	0.468**	0.398**	0.325**
SDCBP2-AS1	-1.6688133	0.01255236	0.364**	0.440**	0.398**
AC018647.2	-1.6419179	0.02526014	0.473**	0.564**	0.495**
AL021707.3	-1.6346771	0.00062354	0.474**	0.414**	0.308**
AC004918.5	-1.6338309	0.00248678	0.343**	0.507**	0.344**
AL512791.1	-1.6289312	0.00848895	0.300**	0.389**	0.330**
ZNF32-AS2	-1.6124161	0.01808374	0.398**	0.505**	0.446**
AL021707.6	-1.5902077	0.01126551	0.491**	0.325**	0.337**
AL031717.1	-1.5894149	0.00164283	0.301**	0.388**	0.340**
AC011498.2	-1.5851675	0.00557956	0.314**	0.428**	0.469**

AC023509.3	-1.5425629	0.00057116	0.453**	0.516**	0.466**
PTOV1-AS2	-1.5370888	0.02882291	0.510**	0.531**	0.541**
ASB16-AS1	-1.5359745	0.04922316	0.513**	0.602**	0.556**
AC005046.2	-1.5336114	0.04907562	0.401**	0.479**	0.387**
PSMG3-AS1	-1.5049124	0.00410143	0.371**	0.488**	0.444**
AC107375.1	-1.4945794	0.04699333	0.327**	0.465**	0.491**
AC108449.2	-1.4904897	0.03052144	0.419**	0.519**	0.431**
AC092329.4	-1.4809441	0.00113002	0.368**	0.319**	0.349**
AP000757.1	-1.4571708	0.00209095	0.455**	0.617**	0.640**
ELF3-AS1	-1.4535845	0.03980619	0.462**	0.533**	0.440**
U73166.1	-1.4299915	0.0293123	0.373**	0.442**	0.365**
AL355102.4	-1.4294246	0.01552802	0.371**	0.438**	0.392**
AP000873.2	-1.3859813	0.00653686	0.356**	0.381**	0.345**
LINC00342	-1.3829663	0.03757091	0.409**	0.445**	0.408**
CD2BP2-DT	-1.3673556	0.04252447	0.412**	0.413**	0.403**
NNT-AS1	-1.3669217	0.0075646	0.483**	0.537**	0.520**
AC109460.2	-1.3447162	0.00387194	0.457**	0.595**	0.480**
TAPT1-AS1	-1.3444711	0.00755066	0.365**	0.442**	0.477**
LINC00174	-1.3422355	0.0475299	0.321**	0.419**	0.501**
AL928654.2	-1.3056958	0.02248803	0.431**	0.475**	0.354**
AC092718.4	-1.2806055	0.00513297	0.404**	0.383**	0.338**
AC012306.2	-1.2577439	0.03181441	0.499**	0.447**	0.479**
AL662844.4	-1.2509253	0.0395717	0.325**	0.377**	0.436**
AC005332.3	-1.23033	0.00345507	0.409**	0.507**	0.462**
ZNF790-AS1	-1.2297593	0.00788757	0.353**	0.411**	0.422**
MNX1-AS2	-1.220973	0.01602276	0.368**	0.518**	0.384**
LINC00909	-1.2134095	0.00966539	0.443**	0.552**	0.532**
PINK1-AS	-1.2082154	0.02926956	0.322**	0.468**	0.375**
LINC00957	-1.2022734	0.0023393	0.414**	0.500**	0.536**
AP002748.4	-1.1831596	0.00326179	0.475**	0.468**	0.373**
MMP25-AS1	-1.1718159	0.02884311	0.534**	0.539**	0.502**
AC005332.6	-1.16633	0.00321236	0.567**	0.702**	0.568**
AC026801.2	-1.1621024	0.0141541	0.310**	0.384**	0.371**
AC026471.1	-1.1603295	0.04653954	0.481**	0.599**	0.467**
CTBP1-DT	-1.1460419	0.02677671	0.489**	0.512**	0.467**
AC091563.1	-1.1425869	0.00202504	0.335**	0.418**	0.422**
TMEM44-AS1	-1.1341856	0.00314751	0.433**	0.484**	0.402**
LINC00667	-1.1263566	0.04061672	0.487**	0.621**	0.558**
EXOC3-AS1	-1.1216311	0.00937724	0.405**	0.461**	0.398**
AC004067.1	-1.0638121	0.01285972	0.346**	0.370**	0.376**
AC008760.1	-1.0593413	0.00243737	0.489**	0.562**	0.567**

ZNF32-AS1	-1.0499742	0.00213492	0.311**	0.416**	0.382**
AL022328.1	-1.0439312	0.01237131	0.347**	0.381**	0.387**
MCM3AP-AS1	-1.0237413	0.04951237	0.348**	0.382**	0.406**
AC106782.5	-1.0111575	0.015448	0.300**	0.409**	0.421**

Supplementary Table 5. Top dysregulated lncRNAs in pancreatic islets derived from *FOXA2*$^{-/-}$ iPSCs in comparison to those derived from WT-iPSCs (p<0.05)

LncRNA	Log2 FC	p-value
**Downregulated**		
AC129926.1	-3.48460498	0.019381821
SOX21-AS1	-3.375206673	0.002300972
CERNA2	-3.262968571	0.008476292
AC023090.1	-3.224804125	0.000457822
UPK1A-AS1	-3.121530334	0.001216183
LINC02864	-2.98476449	0.002945274
ADIRF-AS1	-2.869144331	0.000116201
AL133325.3	-2.737498389	0.000396846
ZNF667-AS1	-2.717560856	0.000210262
AC069277.1	-2.679374923	0.000701003
AP004608.1	-2.592398592	0.000143667
AL132857.2	-2.551431232	0.007867556
AL121761.1	-2.47631697	0.002825188
MIAT	-2.447641254	0.006128212
AC104958.2	-2.388023736	0.003460361
AC083841.1	-2.348229212	0.000573324
AL021392.1	-2.219282366	0.01115918
AC011298.1	-2.214871572	0.002120868
AP001207.3	-2.173815279	0.001849648
AL035661.1	-2.108152808	0.002375826
UNC5B-AS1	-2.002541828	0.012839594
AC011503.2	-1.970352528	9.47E-05
AL390719.2	-1.921193138	0.00881401
AC091563.1	-1.852901184	0.000864753
AC106886.2	-1.819302361	0.008266789
LINC01238_1	-1.793295656	0.007070149
AC103957.2	-1.793070902	0.003296627
AC008115.3	-1.792449559	0.004777938
AC079921.2	-1.781357833	0.00114432
AL445985.1	-1.761223343	0.003421879
AL356234.3	-1.734263019	0.026749026
AC090510.3	-1.724388363	0.001672888
AL035587.3	-1.723283511	0.000992016
AL355312.4	-1.709600214	0.003264729
AC244197.2	-1.701001108	0.004129482
AL670729.3	-1.697013333	0.027547979
MEG3	-1.695537434	8.07E-05
AC009065.3	-1.694795227	0.000919993

AC110619.1	-1.669204248	0.015400098
AC240565.3	-1.666753794	0.031847452
AC016590.3	-1.656663538	0.000219614
AC090181.2	-1.6518749	0.004123116
LINC00261	-1.650881593	9.75E-05
LINC00689	-1.646243973	0.001386848
AL356094.2	-1.638031669	0.00474561
AL645608.7	-1.631756686	0.029206538
U73166.1	-1.570767863	0.007890927
AC009407.1	-1.563563758	0.024661686
AC097639.1	-1.541881067	0.01047655
UCA1	-1.534863852	0.042766936
**Upregulated**		
AC087477.4	3.214562401	1.16E-05
LINC02527	2.888949929	6.94E-05
MIR1-1HG-AS1	2.78635734	7.80E-05
B3GALT5-AS1	2.644371079	7.75E-05
AC108752.1	2.609795709	0.001563061
AC104072.1	2.328946385	0.005477238
AC087477.2	2.217890883	0.000124451
AC073210.3	2.185641978	0.009882493
AC108865.2	2.166235527	0.014912326
PCAT7	2.15746891	0.000806347
FAM224B	1.973445618	0.000903283
AC027031.2	1.903921892	0.027906454
DNM3OS	1.897337772	0.00017246
LINC01896	1.865518686	0.00196593
AC009119.1	1.820332994	0.008370084
AC124067.4	1.790242407	0.000164746
AC022075.1	1.761674942	0.01388671
LINP1	1.736793799	0.006822287
GATA2-AS1	1.720262435	0.00405468
AC111000.4	1.703204637	0.000422955
GACAT2	1.687148422	0.024995129
AC060834.2	1.654983994	0.012771326
CYMP-AS1	1.643936587	0.018928494
PCAT14	1.625853294	0.04664886
AL138479.2	1.561905485	0.002129701
LINC02511	1.550251864	0.011021669
SPATA8	1.545579037	0.000119622
CCDC144NL-AS1	1.538424801	0.001356101
LINC02732	1.501243725	0.020941838
AP005482.2	1.466591464	0.005649426
LINC02253	1.413305897	0.016722866
AC011445.1	1.397484474	0.004259649
HECW2-AS1	1.394404826	0.016064847
KC877982.1	1.37625418	0.020079633
AC099518.5	1.360972707	0.021302903
PANCR	1.326794487	0.048158308
AP001528.1	1.313557309	0.000458221
AL358215.3	1.292869661	0.023233869
AC005082.1	1.290320574	0.000645306
AC098798.1	1.288323092	0.000306955

AL049775.1	1.27009231	0.004692604
AL121578.2	1.263747794	0.020135517
NPSR1-AS1	1.253842645	0.043614394
AF106564.1	1.244162868	0.036997446
AC020909.2	1.230621503	0.013378815
KDM2B-DT	1.205806309	0.017857531
AC011139.1	1.188610383	0.041423128
LINC00648	1.161374652	0.046813604
AL390800.1	1.153916667	0.000321078

Supplementary Table 6. List of downregulated lncRNAs with common strong correlation to FOXA2, PDX1, NKX6.1, and ABCC8 with a Pearson correlation (PC) > 0.3 in the pancreatic islets derived from *FOXA2* $^{-/-}$iPSCs in comparison to those derived from WT-iPSCs (*p*<0.05).

LncRNA	Log2 FC	p-value	PC to FOXA2	PC to PDX1	PC to NKX6.1	PC to ABCC8
ZNF667-AS1	-2.7175609	0.00021026	0.436**	0.587**	0.549**	0.400**
AL390719.2	-1.9211931	0.00881401	0.587**	0.582**	0.419**	0.361**
AC091563.1	-1.8529012	0.00086475	0.335**	0.418**	0.422**	0.391**
AC090510.3	-1.7243884	0.00167289	0.319**	0.370**	0.406**	0.346**
AC097639.1	-1.5418811	0.01047655	0.377**	0.434**	0.356**	0.326**
AC110285.2	-1.5091246	0.00943956	0.313**	0.433**	0.334**	0.330**
AC107464.3	-1.4874602	0.03289592	0.303**	0.400**	0.371**	0.313**
AC016394.2	-1.4490727	0.03173317	0.305**	0.390**	0.433**	0.372**
AC011462.4	-1.4430689	0.00031872	0.355**	0.415**	0.408**	0.328**
ZNF790-AS1	-1.3528114	0.00145091	0.353**	0.411**	0.422**	0.344**
AC006435.2	-1.2727405	0.04433202	0.350**	0.484**	0.500**	0.488**
AL590617.2	-1.1898488	0.00069819	0.322**	0.465**	0.456**	0.384**
RPARP-AS1	-1.1768327	0.00054477	0.497**	0.639**	0.550**	0.433**
CD27-AS1	-1.1406858	0.02874059	0.470**	0.606**	0.599**	0.426**
MNX1-AS2	-1.1191168	0.01121461	0.368**	0.518**	0.384**	0.340**
FAM201A	-1.0741873	0.00589022	0.377**	0.489**	0.504**	0.410**
KMT2E-AS1	-1.0672028	0.01315702	0.361**	0.403**	0.431**	0.332**
AC234582.1	-1.0557378	0.01377192	0.397**	0.470**	0.426**	0.316**
ZNRD2-AS1	-1.0543007	0.00266566	0.424**	0.471**	0.469**	0.384**

SLC25A25-AS1	-1.0534922	0.00894033	0.484**	0.453**	0.441**	0.393**
AC108488.1	-1.0384066	0.04593561	0.466**	0.525**	0.507**	0.413**
AL450998.2	-1.0090053	0.00579397	0.413**	0.561**	0.557**	0.358**
AC006333.1	-1.0062851	0.03861764	0.517**	0.601**	0.532**	0.418**
